# A framework for evolutionary systems biology

**DOI:** 10.1186/1752-0509-3-27

**Published:** 2009-02-24

**Authors:** Laurence Loewe

**Affiliations:** 1 Centre for Systems Biology at Edinburgh, The University of Edinburgh, Darwin building, Kings Buildings, Mayfield Road, Edinburgh, Scotland, EH9 3JU, UK

## Abstract

**Background:**

Many difficult problems in evolutionary genomics are related to mutations that have weak effects on fitness, as the consequences of mutations with large effects are often simple to predict. Current systems biology has accumulated much data on mutations with large effects and can predict the properties of knockout mutants in some systems. However experimental methods are too insensitive to observe small effects.

**Results:**

Here I propose a novel framework that brings together evolutionary theory and current systems biology approaches in order to quantify small effects of mutations and their epistatic interactions *in silico*. Central to this approach is the definition of fitness correlates that can be computed in some current systems biology models employing the rigorous algorithms that are at the core of much work in computational systems biology. The framework exploits synergies between the realism of such models and the need to understand real systems in evolutionary theory. This framework can address many longstanding topics in evolutionary biology by defining various 'levels' of the adaptive landscape. Addressed topics include the distribution of mutational effects on fitness, as well as the nature of advantageous mutations, epistasis and robustness. Combining corresponding parameter estimates with population genetics models raises the possibility of testing evolutionary hypotheses at a new level of realism.

**Conclusion:**

EvoSysBio is expected to lead to a more detailed understanding of the fundamental principles of life by combining knowledge about well-known biological systems from several disciplines. This will benefit both evolutionary theory and current systems biology. Understanding robustness by analysing distributions of mutational effects and epistasis is pivotal for drug design, cancer research, responsible genetic engineering in synthetic biology and many other practical applications.

## Background

Mutations with weak effects on fitness that interact with each other are of great interest to evolutionary genetics and genomics, as their long-term consequences are much harder to predict than those of mutations with large effects. These mutations with small effects are also much more frequent [1,2]. Systems biology has accumulated much data on mutations with relatively large effects by using experimental methods and theoretical tools like flux balance analysis, which analyses the flux of metabolites in biochemical reaction networks [3-12]. For example, flux balance analysis in yeast allows the prediction of the effects of gene knockouts on growth in yeast for about 90% of the genes studied [13-15,9] and this has been used to investigate epistasis [16]. However, mutations with small effects are not easily analysed with stoichiometric modelling techniques like flux balance analysis, and wet-lab observations are usually too insensitive for analysing many effects of interest for questions of long-term stability. Thus kinetic modelling techniques are required. These are frequently based on ordinary differential equations (e.g. metabolic control theory [5,6,17-22,3]) or on stochastic simulations [5,23,24]. New techniques, hybrid approaches and equivalences between existing techniques are constantly being developed (e.g. combine flux balance analysis and metabolic control theory [6]; translate between stochastic simulations and ordinary differential equations [25]; see also [26]). The pace of theoretical and experimental developments raises the possibility that realistic quantitative models of many subsystems of living organisms might become available in the future.

Here I propose to use detailed current systems biology models to analyse distributions of mutational effects and epistatic interactions more rigorously. Such analyses are important for understanding robustness in biological systems and could facilitate the generation of evolutionary hypotheses at a new level, which in turn could provide new insights of interest to current systems biologists. At the heart of such efforts is the construction of realistic and reliable models of the mechanistic realities of life. This new approach is well suited for investigating mutations with very small effects, which are particularly difficult to quantify by other methods. To introduce the new approach I quickly review progress in molecular biology and evolutionary theory separately, before suggesting how both might be combined.

### Current systems biology

Molecular biology has a strong tradition of inferring molecular interactions from well-designed experiments that produce clear-cut results and require little or no quantitative analyses. The success of molecular cell biology and related disciplines has led to the accumulation of so much knowledge that further progress increasingly depends on detailed quantitative models [27,28]. Recognising this, some experimentalists have started to collaborate with theoreticians to develop such models, which has led to the emergence of current systems biology [29-34]. These models aim to capture the essence of important intracellular interactions of the system under investigation. An important goal of systems biology is to discover general principles by developing and using the tools that are needed for analysing models of these interactions [5]. Some go further by working towards ambitious long-term goals such as building a virtual cell [35], a virtual plant [36] or even a virtual human [37], which could then help with designing drugs by predicting undesirable interactions *in silico *([38]. Nobody denies that these goals are far from realisation and some doubt that science will ever get there. However, scientists agree that many important discoveries can be made by working towards these goals, if experimental biologists and quantitative modellers work together [39]. Current systems biology mostly focuses on building and improving more limited models, until predictions match observations from wet-lab experiments in a continuous cycle of (1) experimental observations, (2) theoretical model improvement, (3) quantitative predictions using computers and (4) suggestions for new experiments that help refine models [30,31]. In this paper, 'current systems biology' denotes 'molecular systems biology' along with 'cellular', 'tissue', 'organ' and 'developmental systems biology' in order to capture all quantitative systems biology models that can be used to predict the properties of individuals. This is distinct from 'ecological systems biology' that was popular a few decades ago [40,41] and that used a systems theory approach to investigate how selection shaped the properties of populations in their natural environment.

### Evolutionary theory

Evolutionary biology has a long tradition of mathematical modelling in population genetic theory that frequently abstracts biological details [42-56]. For example, the concept of 'fitness' [45,57-59] is a powerful and widely used abstraction that reduces all molecular, developmental, biochemical, cellular, neuronal, behavioural, physiological and other intra- individual biology to a single number, which can usually be defined as the average number of offspring that will effectively reach the next generation in a certain environment. The concept of a 'selection coefficient' is a similarly successful abstraction, as it simply summarises the effects of a new mutation on fitness. This allows the classification of mutations according to their long-term evolutionary behaviour: deleterious mutations will be selected against and thus never get fixed in a population, effectively neutral mutations will accumulate by random drift as if they had no effect on fitness and advantageous mutations will accumulate faster than neutral ones due to positive selection. While this simple caricature omits the transitions between these extremes, the mathematical theory exists to compute all relevant details. The corresponding population genetics work is one of the scientific successes of the 20^th ^century [44-47,49,50,53-55]. The rigorous nature of many population genetics models and their extensive analysis has lead to key insights in analysing genomic sequences [2,60-64]. The current hunt for functional sequences by scanning genomes for signatures of positive selection frequently uses this framework as well [2,65-67]. In addition, experimental evolution approaches have contributed much to our understanding of evolution, especially in microbes and RNAs that allow 'experimental paleontology' for going back in time and dissecting evolutionary events in detail [68-84]. However, recent work also shows that the simple molecular biological assumptions behind many of the evolutionary analysis methods limit their applicability. For example, not all synonymous mutations are effectively neutral [64,85,86], gene order is not random [87], back-mutations and compensatory mutations can be important [76-78] and epistatic interactions between mutations are frequent [88-92]. Many analyses could be much more rigorous, if the distribution of deleterious and advantageous mutational effects [2,93] were known along with the distribution of epistatic effects [94]. Further progress in analysing evolution will require increasingly realistic models of the underlying molecular interactions.

### A powerful combination

While evolutionary genetics and molecular biology have been very successful in furthering our understanding of the natural world, I propose that combining them even more closely with the help of current systems biology models will significantly improve their power to generate testable hypotheses. The enthusiasm for quantitative descriptions of mechanistic processes in current systems biology could benefit from and contribute to the evolutionary biology objective to understand the forces that shape the existing diversity of life. A functional synthesis of experimental molecular biology and evolutionary biology has been suggested before [95,96]. I propose to add current systems biology models to the combination.

Systems biology can provide maps from genotypes to phenotypes that are much closer to reality than the simple models often used in evolutionary genetics. These maps come in the form of computational models that can allow the automated (and possibly quick) assessment of the effects of a change in the system.

Evolutionary genetics in turn can help analyse effects that are important in the long term, but too small for observation in any laboratory [97,98]. It might also help to identify genetic structures that are no longer optimal due to a relaxation of purifying selection. The molecular functionality of such structures can no longer be assumed to be optimal, as slightly deleterious mutations may have compromised functional integrity [10,99-102]. Quantitative population genetic theories of mutational pressure and genetic drift are powerful tools for analyzing such situations that demonstrate the limits of purely adaptationist assumptions [56,103,104].

**Figure 1:**
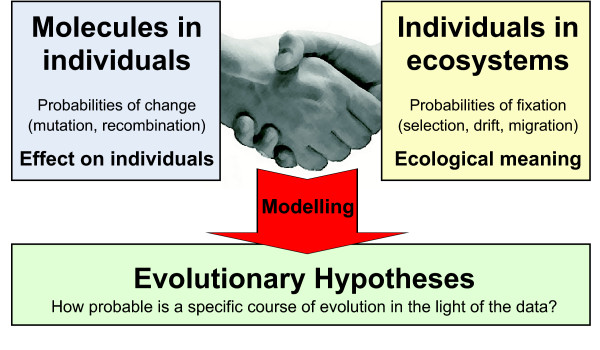
**Evolution has a great potential to unify biology**. The **left box **includes all fields of biology that describe processes within an individual (biochemical, molecular, cell, developmental, neuro, biology) and assumes that they can be integrated into a computable genotype – phenotype map. It also assumes knowledge about mutation and recombination so that probabilities of change from the current genotype to all other possible genotypes can be computed. The **right box **includes all fields of biology that describe processes at the population level and above. This is the place of ecology and of evolutionary processes like selection, genetic drift and migraton. The right box assumes that the fate of new mutations in the population can be tracked so that probabilities of fixation can be computed. **Interactions **between the boxes will exist, but should be much less frequent than interactions within the boxes. For example, the molecular recombination machinery will be important to determine the genotypes of offspring, but its outcome strongly depends on what types of parents are available, which is a population level question. The proposed separation of concerns facilitates clarity of thought about how the modelled processes work.

Much progress in biology depends on the construction of testable null-hypotheses [105]. Bringing together the host of molecular and other information about individual organisms with the wealth of knowledge about evolutionary factors provides the opportunity to develop new testable evolutionary hypotheses. Such a research programme depends on close interactions between the molecular side and the population side of biology (Figure 1). Two recent developments fuel hope for such collaboration.

First, simplistic approaches in both branches of biology are reaching their limits after decades of research. Familiar simplifying assumptions in evolutionary biology are questioned and many researchers are getting increasingly interested in the molecular details of their systems. At the same time molecular biologists increasingly realise that quantitative modelling is actually worth the effort [29,39].

Second, dealing with the flood of *omics data requires new hypotheses. While some seem to doubt the inherent worth of the "new descriptive biology" that has arisen from the massive amounts of high-throughput data, the value of hypothesis-driven research is unquestioned – if it is possible to find interesting questions. The proposed synthesis of evolutionary systems biology is likely to further that by opening up new lines of enquiry, inspired by classical questions in molecular and evolutionary biology.

"*Nothing in biology makes sense except in the light of evolution*" [106] was Dobzhansky's way of highlighting the unifying power of the modern theory of evolution. "*Nothing in evolution makes sense except in light of population genetics*" [101,99] expresses the need for rigorous mechanistic models of evolution, a need that is felt by many evolutionary geneticists. Will it be the case in the future that "*nothing in population genetics makes sense except in the light of systems biology*"? Here is what we can learn from these disparate fields that would contribute to the unified view of biology (Figure 1):

Models at the molecular level can be used to compute the probabilities of accessing particular genotypes by mutation and recombination. Current systems biology models then might compute the key phenotypic properties of the corresponding genotypes. Together these models could predict how far in genotype space an offspring individual can move from its parents and the functional consequences of a given move. They might also allow the computation of an approximate fitness function, which determines the effect of any particular genotypic change on fitness-related properties. The prediction of some phenotypic properties from genetic information and a current systems biology model has recently been shown to be possible for some systems [11,13,15,107-110] and encourages more work in that direction.

Models at the population level could then be used to predict population sizes, population structures and the consequences of resulting genetic drift, migration and selection. Selection might be linked to the phenotypic properties computed by the molecular models by identifying their ecological meaning in terms of survival probabilities and rates of reproduction in specified environments. These models could then compute the fate of new mutations and as a consequence they might predict long-term evolutionary changes for a whole range of systems between single populations and whole ecosystems.

If molecular and population models are combined at a very high level, one can envision the formulation of entirely new mechanistic evolutionary hypotheses. The central role of calibrated computational systems biology models in this approach extends the applicability of this framework beyond that of the functional synthesis of experimental molecular biology and evolution that was proposed elsewhere [95,96,111-113]. Provided enough computing power is available and the models have been constructed carefully, one could test the evolutionary consequences of relevant molecular scenarios *in silico*. I propose to utilise the momentum in current systems biology to lay the foundations for building such high-level models. This should not be prohibitively complicated in systems, where most of the hard work will be done independently by 'traditional' current systems biology research. The hard work will be to produce reasonably accurate mechanistic models of the molecular machinery of some interesting aspect of life. To make such work fruitful for evolutionary systems biology I propose to extend these models so that they can compute 'fitness correlates'.

It may be of interest that historical precedents exist for a successful synthesis of knowledge from systems biology and evolutionary approaches (see Discussion; e.g. [111,114-116]).

### Aims

This article provides a perspective on a new framework that can help bring together evolutionary theory and current systems biology, which have much to offer to each other. Central to this approach is the definition of fitness correlates that can be computed in current systems biology models and that can be calibrated experimentally. Below I first introduce a new way to look at adaptive landscapes that helps to define fitness correlates. Then I discuss how this novel approach can help investigate some longstanding topics in biology that are related to the adaptive landscape. These topics include among others the distribution of mutational effects, epistatic interactions and canalisation that leads to robustness. Finally, a list of challenging questions and some benefits of the new approach for current systems biology are given.

The individual steps that I describe in the framework below have been demonstrated to be achievable in *different *biological systems (see refs). Considerable work will be required to demonstrate for the first time that all steps can be used in combination to better understand the *same *biological system. After that major milestone has been achieved, all methods need to be applied to more biological systems until these analyses become routine work for a wide range of systems in the distant future. The evidence presented here suggests that evolutionary systems biology at that level will eventually become possible.

Evolutionary systems biology will only be successful to the extent that rigorous quantification of its hypotheses can be achieved. Rigorous quantification requires mathematical and statistical frameworks for constructing specific models. It is not the purpose of this paper to define such frameworks in detail (this would fill volumes). I rather intend to provide a perspective that sets the scene for the use of more detailed quantitative frameworks, which will have to be described or reviewed elsewhere. The wide range of disciplines that contribute to evolutionary systems biology makes it impossible to adequately describe the state of the art, so I often limit myself to exemplary references. This paper is not a detailed guide, but rather a rough overview of methods that might be important for evolutionary systems biology with an indication of how they could fit together into the big picture.

## Results

### Adaptive landscapes

An important overarching goal of evolutionary systems biology is to understand and navigate adaptive landscapes. This skill can help solve many practical problems. Adaptive landscapes were first introduced by Wright to facilitate an intuitive understanding of basic properties of the evolutionary process [117-119] and could play a pivotal role in closing the gap between microevolution and macroevolution [120,121]. Depending on which aspect is being emphasised, these landscapes (or surfaces) are also called selective landscapes, fitness landscapes [118,122,123], phenotype landscapes [124-126] or mutational landscapes [127,128,69,68]. Historically, landscapes have been defined in three ways, which differ in their understanding of the *plane*: Wright's landscape of individual genotypes [117-119], Wright's landscape of genotype frequencies [119,118] and Simpson's Landscape of phenotypic properties that was later formalised by Lande [119,129-137].

Another popular model for understanding adaptive evolution is Fisher's geometric model of adaptation [45,138-144]. In this model a multidimensional plane is defined by quantitative traits and mutations are often expected to change several traits at once in random directions, facilitating adaptive walks to the optimum. True to Fisher's original presentation, the geometric model of adaptation is rarely visualised as an adaptive landscape despite the underlying conceptual similarities.

Before I break down adaptive landscapes into different levels below, some common features of all adaptive landscapes need to be reviewed. Each adaptive landscape is intrinsically linked to a replicating unit that experiences selection. Fitness is measured from the perspective of that unit, which is usually an individual, but could also be a replicating cell (e.g. cancer) or group (e.g. beehive) [145-148]. For the moment we will focus on 'non-nested' adaptive landscapes, where only one type of replicating unit is considered (see level 3 below for exceptions).

#### Common features

Like in geographical landscapes, adaptive landscapes have a *plane *that determines all possible places in the landscape and a *height *that is associated with each point in the *plane*. It is possible for objects to 'move' in the *plane *(e.g. by mutation, recombination), but moving is usually somehow restricted. Local topology determines whether movements result in a change of *height*. Adaptive landscapes differ from geographical landscapes in the way *plane*, *height *and the moving of objects are defined:

• ***Plane ***= **'genotypes'**. Depending on the level (see below), the *plane *can be defined directly in terms of genotypes or indirectly by phenotypic traits at the molecular, organismal or population level, assuming that these traits are ultimately determined by genotypes. Since organisms are complex, the *plane *is usually a high-dimensional space with very non-intuitive properties and complicated restrictions on 'movements'.

• ***Height ***= **'fitness'**. The *height *can be either a direct population genetic measure of fitness or some lower-level phenotypic property of interest that might be indirectly related to fitness. Population genetic measures of fitness ideally average over all possible scenarios, combining their weighted contributions to some rigorously defined measure of fitness like 'reproductive value' [58] or 'inclusive fitness' [149]. Fitness definitions can be complicated by the fact that the most important long-term measure of fitness, the ability to contribute genetic material to the next generation, depends on the properties of other individuals in the population. Thus computations of *height *can range from very simple to very complex, depending on the model. Lower levels of the adaptive landscape often allow for different ways of defining *height *or require a combination of many properties to define *height*. Technically this leads to many corresponding landscapes with a shared plane. It is usually desirable to combine such different *heights *into a '*height*-vector', which simplifies the abstract treatment, even though it is no longer easy to visualise.

• **Objects **are defined by a position in the *plane *that is associated with a *height*. The identity of objects depends on the *level *of the adaptive landscape under consideration (see below). The *plane *for an object at a higher *level *may consist of a whole array of objects at a lower *level*. This is particularly apparent at *level *7, where each object is a whole population of individuals.

• **Environmental changes **clearly affect subsequent adaptive walks on the landscape and can be seen as part of a selective regime, which averages over all relevant environments. There are two ways of including environmental variation in the adaptive landscape; one adds environmental parameters as dimensions to the *plane*, while the other adds them as dimensions to the *height*-vector. Both appear to be conceptually equivalent if all *levels *of the adaptive landscape are defined consistently. The preferred approach may vary with the model.

For a population of objects in a constant environment to move on this landscape, new genotypes need to be produced by mutation and recombination or be imported by migration. In such a setting each population will be 'pushed' uphill by selection if (i) sufficient time is available, (ii) the height is correlated with the ability to contribute genetic material to the next generation, for example, by better survival and (iii) the gradient is steep enough to overcome the potentially opposing effects of non-selective forces.

**Figure 2:**
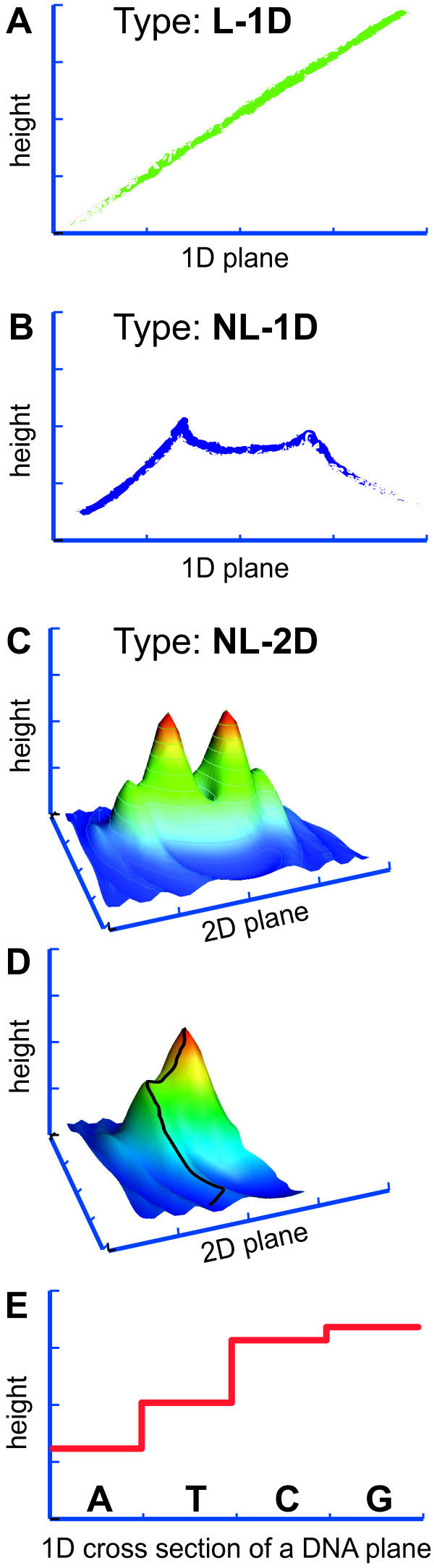
**Examples of simple *types *of adaptive landscapes**. **(A)**–**(C) **give examples for the complexity of the corresponding *type *of adaptive landscape. **(D) **illustrates the principle that adaptive walks on high-dimensional landscapes reaching a local optimum in some dimension may continue to even higher peaks by optimising other dimensions – if genetic correlations allow this and the relevant parts of the landscape remain constant for long enough. In this example, the black line denotes a hypothetical adaptive walk, which follows the steepest ascent to a first saddle point on the blue ridge, then continues to optimise by changing direction to follow that ridge until it reaches a second saddle point on the green ridge, only to change again directions before reaching its optimum in that landscape. For such a scenario, this landscape must be independent of environmental or other changes during the adaptive walk and new mutations must be capable of producing individuals that represent random steps in that landscape. These random steps can be achieved by sequential steps in different dimensions, if reciprocal sign epistasis does not prohibit this [91]. **(E) **illustrates how a cross section of the most fine-grained adaptive landscape might look like. Here each dimension corresponds to one functional DNA sequence position in the genome. The number of possible steps within each such dimension is small, even if the example given is extended to include the absence of the base and epigenetically methylated bases. In such landscapes the simplicity of options within one dimension is countered by an extraordinary complexity of epistatic interactions between dimensions. All landscapes shown are completely arbitrary and serve only illustrative purposes. See the main text for a guide to the nomenclature of *types *of adaptive landscapes and the various definitions of *height *('fitness' or traits that are correlated with it) and *plane *('genotypes' or traits encoded by them). The latter two depend on the *level *of the adaptive landscape.

The adaptive landscape conveys a very powerful image of the evolutionary process that is frequently referred to in biology [119,134]. Unfortunately, its complexity and non-intuitive features make it difficult to use, even if environmental changes are ignored. This has led to various criticisms of the concept (see reviews in [119,134,150]). The occasionally interchangeable use of the three historic ways of defining the landscape contributed to the confusion [119]. The following features of adaptive landscapes are particular non-intuitive:

• ***Plane *dimensionality**. Humans have difficulties visualising more than three spatial dimensions. Yet realistic adaptive landscapes can have dozens to hundreds of dimensions if defined in terms of quantitative traits and many millions if defined in terms of functional DNA sequence sites. In the light of this enormous gap of dimensionality, it rarely matters whether dimensions are collapsed into one or two dimensions for visual purposes, as such images will be misleading in either way (see examples in Figure 2). Mathematical representations do not suffer from this limitation, if their level of abstraction can be justified biologically.

• **Restricted movements**. Due to the complicated functional network that underpins the *plane*, movements by mutation or recombination to a new point in the plane are not easily predicted in *planes *of phenotypic values (such as shown in Figure 2C). Such movements are easily predicted in a *plane *of genotypes, but then mutation can only move in one (or a few) out of many dimensions of the *plane *in any given generation (Figure 2E). This is counter-intuitive, when compared to the geographical 2D landscapes that humans are used to and that usually allow steps in arbitrary directions. A situation where no restrictions on movements exist can be approximated by a situation where movements are restricted to one dimension per generation, if reciprocal sign epistasis [91] does not produce 'fitness-valleys' that could have the potential to block a particular adaptive walk. Thus overall movements in the *plane *can be restricted in unexpected ways.

• **Fitness as *height ***might suggest high mountains for large reproductive capacities. However, the effective number of offspring produced by most individuals is about *one*, since most population sizes stay approximately constant over long periods of time. Density-dependent competition and limited resources will adjust absolute numbers of offspring accordingly. In most situations, selection acts upon slight relative differences in a population. Defining height as 'reproductive value' [58] or 'inclusive fitness' [149] solves these problems, but can be mathematically challenging [59].

To make the difficulties with dimension reduction explicit and facilitate discussions of the "Linear Fitness Correlates Hypothesis" described below, it is helpful to distinguish different *types *of landscapes.

#### Different *types *of adaptive landscapes

The following nomenclature can be applied to adaptive landscapes at all *levels*. To distinguish different landscape *types*, the following three properties can be used (Table 1):

**Table 1:** Properties that define the *type *of an adaptive landscape

**Symbol**	**Meaning**
**L **or **NL**	Is the landscape **Linear **or **Non-Linear**?
**1D, 2D, ..., nD**	How many **Dimensions **are in the *plane *of the landscape?
**C **or **V**	Is the environment **Constant **or **Variable**?

• **Linearity (L) and Non-Linearity (NL)**. In L-landscapes the *height *is a direct linear function of the position in the *plane*, making extrapolations easy. In the much more frequent NL-landscapes, predictions of height are difficult due to the non-linearity that may or may not allow for extrapolations. A more restricted plane within a NL-landscape can have L-landscape properties.

• **Dimensionality (1D, 2D, 3D, ... nD) **determines how many properties define a point in the *plane*. If no dimensionality is specified, 1 dimension is assumed. 'nD' is equivalent to an unknown number of dimensions. It is not possible to model trade-offs between different properties in 1D landscapes, as there is only one property in the plane. All other landscapes can potentially trade-off different dimensions to maximise *height*. If the dimensionality is given by the number of functional base pairs in the genome, then movements on the *plane *are very simple as each dimension can only adopt few points (Figure 2E). However, other dimensions impact the *height *of these points in non-trivial ways.

• **Constant (C) or variable (V) environments**. Since some fitness correlates depend on the environment, it is desirable to specify, whether the environment remains constant or varies with time. If nothing is specified, constant environments are assumed. Additional dimensions of the *plane *or *height *can capture varying environmental properties by changing according to special rules that implement the environmental changes.

Examples of simple *types *of landscapes are shown in Figure 2. The simplest possible landscape is denoted by 'L' (equivalent to 'L-1D-C'). Usually the most complicated (and realistic) landscapes belong to *type *'NL-nD-V'. 'L'-landscapes with high dimensional *planes *are a special case as one can easily define an equivalent '1D' *plane*. Similarly one could use Principal Component Analysis [151] to reduce the dimensionality of 'nD' landscapes, if some dimensions in them combine linearly. To quantify adaptive landscapes rigorously, all relevant dimensions must be either included or kept at a constant value. A relevant dimension is defined as a genotypic, phenotypic or environmental property that affects the *height *of the landscape. Dimensions that do not affect *height *can be ignored. The general properties and *types *of landscapes defined above facilitate the discussion of several *levels *of concrete adaptive landscapes that can be connected to observed data.

#### Adaptive landscapes at seven *levels*

Many discussions of adaptive landscapes prefer to focus on the 'big picture' that defines fitness as the *height *without specifying the *plane *precisely. This is not only confusing [119,118], but also frustrates any attempt to estimate landscapes from empirical data. To facilitate the precise quantification of adaptive landscapes, a quantitative genetics framework has been developed by Lande et al. [129-137]. This framework defines the *height *as the mean fitness of populations and assigns phenotypic properties to the dimensions of the *plane*. It allows the measurement of phenotypic selection in the wild [132,133,152], but does not facilitate the incorporation of molecular functional data [136,144] and depends on phenotypic traits following approximately a Normal distribution after an appropriate transformation [136,137]. Building on Lande's approach, Arnold used path analysis to decompose fitness into fitness components that are determined by functional phenotypic traits [120,153-155]. A central component in this approach is the so-called 'G-matrix' that measures the additive genetic variance and covariance of phenotypic traits encoded by many genes. The G-matrix could be used to predict evolution if the evolutionary dynamics of the G-matrix were known, a problem too complex for existing analytic theory [155]. A potential way forward could be to integrate these quantitative genetics approaches with the various molecular and current systems biology *levels *of the adaptive landscape described below. Indeed, to connect adaptive landscapes to observable molecular functional data, recent work has considered the adaptive landscapes of single proteins and more complex molecular systems [91,156,157,112,95].

The ideal connection of an adaptive landscape to biological data would predict the *height *by *ab initio *calculations from observed data and then compare predicted and observed *heights*. To subdivide this extraordinarily difficult problem into smaller (but still formidable) tasks, I define different *levels *of the adaptive landscape, each with its own *height *and *plane *definitions (Table 2). To resynthesise the big picture from these *levels*, one needs to combine all *heights *of each lower-level landscape to define a point in the *plane *of the corresponding higher-level landscape. Mathematically speaking, each level is defined as a function that computes the *height *for many points in the *plane*, where each dimension corresponds to a parameter. Thus for each level:

**Table 2:** Points on different *levels *of the adaptive landscape

**Level**	**Height^1^**	in the	**Plane^1^**
1	A molecular structure	in the	space of genotypes
2	A molecular function	in the	space of molecular structures
3	A computable emergent property^2^	in the	space of molecular functions
4	A computable fitness correlate	in the	space of computable emergent properties
5	An observable fitness correlate^3^	in the	space of computable fitness correlates
6	The fitness of an individual	in the	space of observable fitness correlates
7	The mean fitness of a population	in the	space of the fitness values of all individuals in the population^4^

^1 ^The *height *and *plane *of a point on each *level *can have one or many dimensions.

^2 ^This *level *can have an arbitrary number of sublevels reflecting the hierarchical nature of biological systems. For example 3.1 could compute cell properties based on molecular functions, 3.2 tissue properties based on cell properties, 3.3 organ properties and so on, as needed.

^3 ^This *level *is a simple 1:1 mapping, if the LFCH can be accepted. In all other cases it may be used to provide some heuristic quantitative link between observed and predicted fitness correlates.

^4 ^The *plane *has as many dimensions as there are individuals in the population. Providing additional information on genotypes or phenotypes allows a massive reduction of dimensions, producing the traditional views of the adaptive landscape that show the mean of a population in genotype frequency space or phenotypic trait space.

*height *= *f *(*plane*)

Combining two *levels *often requires many evaluations of *heights *at the lower *level *to define the *plane *of the higher *level *(subscripts denote *levels*):

*height*_2 _= *f*_2 _(many *f*_1 _(*plane*_1_))

Since the mathematical formalisms can handle many dimensions in principle, no information is lost, even if it is not possible to visualise the landscapes. When defining such formalisms, one must ensure compatibility between lower-level output and higher-level input. Ignoring environmental changes for the moment, I propose the following seven *levels *of adaptive landscapes:

##### 1. A molecular structure in the space of genotypes

**n-dim *plane***: genotype or DNA sequence space with *n *loci.

**m *heights***: deviation of *m *crucial features from the presumably optimal wild type structure or a whole collection of *m *measures that describe the 3D structure.

**Key question**: How do DNA sequence changes influence the structure of macromolecules?

**Data**: Crystallographic structures of wild types and mutants (see ) and comparative modelling of 3D structures in the computer [158,159] provide easy access to the structures of many macromolecules.

**Successes**: General knowledge about mutational effects on proteins [160,161] and structural predictions have been used successfully to detect deleterious mutations [162,163].

**Limits**: If sequences differ by about 50% or more from an experimentally known structure, comparative modelling in the computer becomes increasingly difficult [159]. *Ab initio *modelling remains very challenging, despite decades of research [164]. No insight into the relative importance of mutations in different genes can be obtained.

**Outlook**: A combination of experiments, *ab initio *modelling and comparative modelling will lead to even more confident prediction tools. If only approximate functional rates are required, then experimental methods can provide a shortcut through this and the next *level *(see next *level*).

##### 2. A molecular function in the space of molecular structures

**n-dim *plane***: discrete molecular structures as determined at *level *1.

**m *heights***: *m *different molecular functions of interest (e.g. enzymatic rates).

**Key question**: How does the structure of macromolecules affect their function?

**Data**: Direct predictions of functions from structures [165-167] have been developed only recently for proteins using computational methods that build on experimental data. Generally, databases of kinetic measurements [168,169] are growing and if functional effects of mutations are large enough, they can be measured in experiments or observed while evolving *in vitro *[170-179]. It is also possible to observe protein functions in the form of aggregated rate laws that measure the speed of a group of reactions and can be used to narrow the range of plausible parameters for individual reactions by computational analyses [180]. Research into structure-function relationships and protein engineering [161] has matured to the point where some functional properties are amendable by engineering [113,181]. Mutation accumulation experiments can be used to assess the impact of spontaneous mutations on gene regulation [182]. Having additional copies of genes might affect the intracellular concentration of their proteins [115,183-185] and possibly also metabolic flux [186,187].

**Successes**: In principle it is now possible to extrapolate from known kinetic rates and known protein structures to unknown kinetic rates that employ the same functional mechanism [167].

**Limits**: If *ab initio *predictions of molecular structures are challenging [164], they are even more so for molecular functions. The new comparative methods have not yet been tested in many different systems.

**Outlook**: Experimental methods allow shortcutting of this and the previous level by providing a direct kinetic measurement associated with a known sequence [168-177], although very small differences can be impossible to distinguish. The combination of proteomics techniques with the knowledge of reaction networks promises the estimation of a credible range of individual reaction rates for many enzymes from the observation of aggregated rate laws [180]. Progress on computational methods is impressive [165-167] and could lead to the possibility of routinely predicting small mutational effects on function with some confidence. Growing knowledge in protein design will lead to more confidence in understanding adaptive landscapes at this level [91,161,181]. It is currently not clear, whether computational or experimental approaches will be more efficient in addressing the very hard problem of obtaining kinetic parameters on a massive scale.

##### 3. A computable emergent property in the space of molecular functions

This level is special as it could also be seen as encapsulating many more fine-grained sublevels that mirror the hierarchical organisation of many organisms. For example, molecular functions affect the properties of a cell, which affect the properties of a tissue, which affect the properties of an organ, which affect the properties of an organism (which affect the fitness correlates in level 4). The best choice of sublevels depends on the structure of the multi-level systems biology models considered (e.g. root growth [188], heart [189-191]). If the primary adaptive landscape under investigation depends on lower-level units of replication [148] with their own adaptive landscapes, then these can be accommodated as additional sublevels here. Such 'nested landscapes' help, for example, understanding the conflicts of selection in cancer [192,146].

**n-dim *plane***: *n *molecular functions of many different molecules (from level 2).

**m *heights***: *m *different emergent properties of the biological system (e.g. timing or probability of activities; reliability or mechanical properties of structures; any other conceivable property of an organism or one of its biological substructures).

**Key question**: How do changes in macromolecular function affect the emergent properties of the whole system?

**Data**: The computing of systemic functions is the goal of systems biology modelling, hence many such models have been constructed recently [11,13,31,108,193-198]. Some of their emergent properties can be determined experimentally [11,13,108,195] and can be used to improve the models. Some biochemical networks have a special function during development and their analysis has become increasingly mechanistic (e.g. [199-201]). The realisation of the importance of such networks for the evolution of morphological features has fuelled the rise of 'evo-devo', which combines evolutionary biology and developmental biology [124-126,202-211]. The quality of all computational models at this level is important for further analyses that build on corresponding output. Quality here is hard to measure but will mostly reflect the quantitative accuracy, which in many cases requires the completeness of the mechanistic model.

**Successes**: It is easy to test the sensitivity of many systems biology models with regard to changes in various molecular kinetic parameters. Comparative analyses have shown that some universal properties might exist [194]. Experimental confirmation of some predictions are possible [11,13,108,195,9]. Successful modelling has been achieved in systems as diverse as metabolic reaction systems [9] and developmental modules [199-201].

**Limits**: Computational complexity and poorly known parameters frequently limit the accuracy of computational systems biology models [26].

**Outlook**: Excitement about and investments in current systems biology [212,213] provide reason for hoping that many more high quality systems biology models will be developed to serve as a basis for predicting the emergent properties of molecular, tissue and organismal systems.

##### 4. A computable fitness correlate in the space of emergent properties

**n-dim *plane***: *n *different emergent properties of the biological system (from level 3). These can also be seen as quantitative traits.

**m *heights***: *m *computable fitness correlates of the biological system as predicted for well specified environments (e.g. survival, fecundity, growth rates).

**Key question**: How do observable fitness correlates depend on other emergent properties of the system? The goal is to define computable fitness correlates that are directly proportional to observable fitness correlates.

**Data**: A functional understanding of the system and the mechanistic basis for observable fitness correlates serves as the basis for defining this *level *of the adaptive landscape. Such understanding was experimentally confirmed in some systems [13,108,143,9] (see discussion of fitness correlates below).

**Independent theory**: A longstanding question in evolutionary theory has been, how fitness depends on various quantitative traits that could be viewed as dimensions in the emerging-property-space. A rich body of quantitative genetics theory has been developed to predict fitness effects from changes in an underlying multi-dimensional adaptive quantitative trait space [45,138-142,156,214,120,129-137,153-155]. Despite the absence of detailed biochemical information, such work can have experimental predictive power [143], might infer the effective number of 'molecular phenotypes' of a gene from DNA sequences [156] and could be used to decompose fitness correlates into functional components [120,153-155]. Advances in quantitative genetics methods also allow the estimation of selection on fitness correlates in the wild [152] and the identification of quantitative trait loci if their impact on phenotypic properties is large enough [144]. Such work does not require a mechanistic understanding of the traits as would be gained from quantifying *levels *(1) – (3) above. While this limits the direct applicability of quantitative genetics approaches, one could use the experience with quantitative traits to inspire the definition of computable fitness correlates.

**Successes**: Computable fitness correlates can be defined in metabolic networks with the help of flux balance analysis models [9] and in circadian clocks using other approaches [196]. The former are supported by experiments [13,16,108,9]. Observations also confirm predictions from abstract general models that map quantitative traits to fitness [143].

**Limits**: To provide a good mapping of the adaptive landscape at this level, one either needs a thorough mechanistic understanding of the corresponding fitness correlates or a firm grasp of a general theory that allows for reasonable predictions in the presence of many poorly known interactions. Neither may be easy to obtain for some systems. Testing the accuracy of a given mapping with the help of the Linear Fitness Correlate Hypothesis (see below) can inspire research towards obtaining better mappings.

**Outlook**: The most difficult groundwork for this step is the availability of good computational systems biology models. Defining computational fitness correlates for these models is usually only a minor addition that is based on biological intuition. Once such work has been pioneered for particular types of systems, patterns are likely to emerge. The computational nature of these models makes it easy to analyse very small effects and thus provides an empirical foundation for theoretical analyses that otherwise have to make many non testable assumptions. It will be interesting to see how much of the independently developed quantitative genetics theory that maps quantitative traits to fitness will be confirmed by mechanistically explicit adaptive landscapes of this *level*.

##### 5. An observable fitness correlate in the space of computable fitness correlates

The purpose of this *level *is to test the Linear Fitness Correlate Hypothesis (LFCH) and to make heuristic quantitative adjustments, if computed and observed fitness correlate differences do not match.

**n-dim *plane***: *n *different computable fitness correlates (from level 4).

**m *heights***: *m *observable fitness correlates of the biological system (e.g. survival, fecundity, growth rates). These have to be observed experimentally to calibrate the computational fitness correlates. Ideally, *m *= *n*.

**Key question**: Does the computational model reflect biological reality? If yes, both fitness correlates should be proportional to each other, resulting in a landscape of *type *'L-1D' (see Figure 2A + 3). Experiments with many well-characterised mutants will be required to detect deviations from a 'L-1D' landscape.

**Figure 3:**
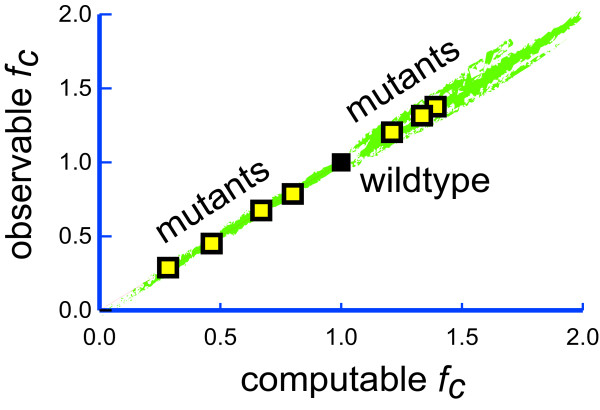
**The Linear Fitness Correlate Hypothesis**. This hypothesis states that it is possible to define a computable fitness correlate (*f*_*cc*_) based on a comprehensive systems biology model that is proportional to a particular observable fitness correlate (*f*_*co*_) like survival, fecundity or growth rate. The resulting adaptive landscape is of type 'L-1D' (see Figure 2). Mutants (yellow squares) with values below the wildtype value can be constructed by introducing deleterious mutations of known effects. Mutants with values above the wildtype can be difficult to obtain in natural environments for fitness correlates that closely follow fitness (the wild type is optimised for these). Artificial environments can solve this problem, as wild types are less adapted here, leaving more room for optimisation. Once calibrated by such mutants, *in silico *estimates can capture very small effects more precisely than direct observations with their accompanying experimental errors. See text for more explanations.

**Data**: Computational fitness correlates data depends on the successful completion of *level *4. Observable fitness correlates data can be readily generated by well-established experimental protocols for measuring properties such as survival, fecundity, or growth rates. The challenge is to find mutants that differ enough from the wild type to result in significant observable differences *and *that are characterised well enough at the molecular level to allow the prediction of computational fitness correlates.

**Successes**: Computational fitness correlates that match experimental observations include the effects of lethal gene knockouts on growth rates in yeast that can be predicted from flux balance analysis in over 90% of all cases [11,13,14,9]. It was also possible to predict epistatic effects [16,143] and adaptive evolution [108] in microbes. Two out of four tested genotypes of a bacteriophage were in moderate agreement with computational predictions of growth rates [109].

**Limits**: The large efforts required for adding a new point to the calibration in Figure 3 can prohibit the screening of enough mutants for reliable calibration in many systems. The approach presented here is new, so experience is sparse. See more details below.

**Outlook**: Comparing observed and computationally predicted fitness correlates is a key aim of evolutionary systems biology. The increasing numbers of quantitative systems biology models with experimental support that are under development will provide increasing opportunities for comparing observed and computed fitness correlates. Computing a specific fitness correlate in a specific system through all levels described above and obtaining a reasonable match with experiments can be seen as evolutionary systems biology's equivalent of sequencing a genome: it was thought to be impossible for a long time, was eventually reduced to a technical challenge and is now done routinely in many labs.

##### 6. The fitness of an individual in the space of observable fitness correlates

**n-dim *plane***: *n *different observable fitness correlates (from *level *5).

***height***: population genetically relevant long-term fitness of an individual. Usually '1D', can be 'nD' if different environments are treated separately.

**Key question**: How much do fitness correlates contribute to evolutionary long-term success?

**Data, Successes, Limits, Outlook**: Life-history evolution models have been used for a long time to address core questions of the adaptive landscape at this *level*, so a rich set of existing theory can be used [132,152,215-217]. This also includes the contributions of the formal Darwinism project [59] that rigorously defines 'reproductive value' as the maximand of evolution [58] (or 'inclusive fitness', if social evolution is considered [149]). If the *plane *of fitness correlates is substituted by the underlying plane of genotypes, then this level becomes equivalent to the first definition of an adaptive landscape given by Wright [117-119]. The prospect of rigorously computing all *levels *up to this one in real biological systems is exciting for everybody with an interest in the integration of biological knowledge.

##### 7. The mean fitness of a population in the space of the fitness values of all individuals in the population

**n-dim *plane***: fitness values (level 6) of *n *individuals in the population. To allow for meaningful analyses, some additional information about each individual is usually given too (e.g. genotype or phenotype). This technically multiplies the dimensions of the *plane *by the number of state dimensions given for each individual. However this also allows for a massive reduction of dimensionality, if only the mean value of the population is of interest for each state dimension.

***height***: average fitness of the population (dimensionality as in *height *of level 6).

**Key question**: What is the population doing as a whole? Are there cases of balancing or frequency-dependent selection?

**Data, Successes, Limits, Outlook**: This *level *provides the link to traditional adaptive landscape representations showing the mean of a population in the space of allele frequencies or in the space of phenotypic traits. The former goes back to Wright [119,118] and the latter to Simpson and Lande [129,134]. While evolution will maximise the mean fitness of a population under standard assumptions, some conditions like non-random mating or frequency-dependent selection may not increase mean fitness, which has led to criticism of this representation of the adaptive landscape (for a review, see [134]). However in many cases quantitative analyses of the population mean are an excellent tool for investigating the adaptive landscape at a high level [118,134,135]. It would be a powerful demonstration of the unifying potential of evolutionary theory, if the classic quantitative genetics analyses of adaptive landscapes could one day be combined with current systems biology models for analysing mutational effects that have so far been too small for direct observations.

In principle each of these *levels *can belong to all *types *of landscapes described before, although non-linear landscapes will strongly dominate some *levels*. Some landscapes will assume a constant environment, which cannot be considered realistic in all cases. Other landscapes are dynamic in that they change with environmental conditions [134,135,218]. To account for changes in the environment, one can either extend the corresponding *planes *or *height*-vectors by additional dimensions (see above).

Since adaptive landscapes are very difficult to visualise, biologists have developed simplifications that focus on particular aspects. Below I will show how two such simplifications can be investigated with the help of fitness correlates. *Distributions of mutational effects *can be derived from adaptive landscapes by choosing a particular reference point and then constructing a histogram of all fitness differences that can be reached from the reference point in a single mutational step. *Distributions of epistatic effects *can be obtained by exploring how much the effects of combinations of multiple mutational steps will deviate from the expectation that all effects are independent. Thus fitness correlates allow the biologically informed investigation of many longstanding questions in evolutionary biology, including the fraction of advantageous and compensatory mutations.

### Fitness correlates

The careful reader will have noticed that adaptive landscapes at *level *5 above are redundant under the ideal conditions of perfect knowledge, where computed and observed fitness correlates are identical. Since we are far from perfect knowledge in many systems, this level is deliberately left in the hierarchy in order to:

• Allow for testing how close one is to perfect knowledge of the system, where the Linear Fitness Correlate Hypothesis becomes true (see below).

• Allow for empirical corrections at *level *5, based on interpolations from experiments with well-characterised mutants in cases where knowledge is less than perfect.

#### The Linear Fitness Correlate Hypothesis (LFCH)

The LFCH assumes that it is possible to understand biological systems mechanistically and states that *a biological system has been understood, once it is possible to define computable fitness correlates that are proportional to observable fitness correlates in corresponding mutants*. In other words, the adaptive landscape at level 5 must be of type 'L-1D' (Figure 2A + 3) for each fitness correlate that is studied. In order to test the LFCH, one has to construct as many mutants as possible with the following properties:

1. Mutants must be well enough characterised to allow prediction of their computable fitness correlates.

2. It must be possible to measure the corresponding observable fitness correlate in the mutants.

3. The observable fitness correlate of the mutant must show statistically significant differences from the reference wild type.

4. Some mutants' fitness correlates should be lower than those of the wild type, while others should be higher to guarantee that values on both sides of the wild type are on the same line (see Figure 3).

#### Fitness decreasing mutants

The construction of mutants with fitness correlate values below the wild type might be achieved by (i) knocking-out genes, (ii) adjusting their regulation or (iii) targeted protein design. Decreasing fitness is expected to be relatively easy, since the wild type is probably close to its evolutionary optimum. The fitness effects of knock-out mutants have been measured on a large scale in yeast [13-15,219,9]. Work that links particular genotypes to particular fitness values is also possible for more complex organisms (e.g. ([220]).

#### Fitness increasing mutants

To obtain fitness values above the wild type is more challenging, because organisms are usually well adapted to their environment. Nevertheless, examples show that this is possible. A large-scale screen that introduced new network connections in the regulatory network of *E. coli *found some of the changes to be advantageous for fitness in a laboratory environment [221]. Another screen that deleted genes from *Bacillus subtilis *found that some deletions actually increased metabolic flux and hence growth rate under some conditions, albeit these deleted genes were important under other conditions [222]. Thus one might frequently have to rely on artificial environments to identify mutations that exceed wild type fitness correlates, since wild types were not selected in these environments and thus cannot be expected to be optimal. As trade-offs are frequent in life history evolution there might be many opportunities for quantifying mutants that exceed wild type fitness correlates. Since ultimate fitness is predicted at a higher level of the adaptive landscape than fitness correlates, it should be possible to test the LFCH on both sides of a wild type value even though wild types are usually optimally adapted to the wild. For a given set of changes in the environment it was possible to predict *in silico *the observable adaptive evolution of *E. coli *[108]. Other experiments observed adaptation to a new environment on the long-term [71-75,223,224]. Some systems might allow the use of biotechnological approaches to increase fitness correlates [225]. Finally, one might consider computational searches to speed up the process (investigating the systems biology model used for this work could provide hints for promising candidates for mutagenesis).

#### When to accept the LFCH?

To thoroughly test the LFCH requires the construction of many appropriate mutants. Technically, the LFCH will never be proven correct for any system, since one cannot categorically exclude that some future mutant will contradict a current L-1D landscape. However, as soon as 3 or more significantly different genotypes line up as expected in this landscape, some empirical support for the LFCH can be said to exist. Obviously many more mutants will be required to increase trust in the understanding of any particular system. A quick (and risky) way of building trust in the current understanding is the experimental confirmation of 'daring' predictions of unexpected and previously unknown properties of the system. Currently, a substantial level of trust exists for flux balance analysis models of *E. coli *[108,7]; a small-scale comparison of 6 predicted and observed non-lethal *E. coli *knock-out growth rates showed a high correlation of around 0.8[12]. In yeast FBA models predict lethal knock-out effects with an accuracy of over 90% [13-15,219,9], where the biomass production flux is the computed fitness correlate and growth rate is the observed fitness correlate. However, there is currently no strong correlation between the predicted and observed growth rate of non-lethal knock-outs known in yeast (FBA usually predicts either very small effects like < 0.1% or approximate lethality; few predictions are on the order of 1%–50% where growth rates would be easy to measure; B. Papp, personal communication). This may be due to limitations of FBA [10] or due to the choice of the underlying optimisation procedures [7,8]. It demonstrates further scope for improvement of the FBA approach.

#### Why not predict fitness correlates directly?

The direct *ab initio *prediction of the absolute values of observable fitness correlates requires a sufficiently comprehensive model of the organism, as many systems influence fitness correlates such as survival or fecundity. In other words, *every *significant sub-system of an organism that affects the traits of interest has to be included, lest the absolute magnitude will be wrong. Not so under the LFCH. Here it is only necessary that the prediction is linear to the observations, as slope and axis intercept can be easily estimated from the observations. This implies that one only needs to compute all the interactions within a given subsystem, while all other independent subsystems can be ignored. Functional knowledge and biological intuition can thus be used to 'divide and conquer' complexity, harnessing the power of evolutionary systems biology for much smaller systems.

#### Limited LFCH support and failure of the LFCH

Many biologists are used to extraordinary noise in observed datasets and the complexity of the analyses required for testing the LFCH suggest that initial results will be very noisy as well. The LFCH can be rejected, if there is enough statistical evidence to reject a linear correlation. This has to be distinguished from situations where there is only limited support for the LFCH. This is the case, when (i) non-linear parts of the *plane *are consciously excluded or (ii) it is not known how to exclude non-linear outliers throughout the *plane*. A few percent of current flux balance analysis predictions are wrong [9,13-15]. Does that justify rejection of the LFCH? As long as all relevant results are reported, it will probably remain a matter of personal taste as to what cut-off levels will be used to "accept" or "reject" the LFCH – similar to current use of P-values in hypothesis testing. Any limitation of support for the LFCH obviously indicates that there is further room for improving the model.

#### Calibration

Technically, one can use an assumed linear relationship for the initial calibration of computed fitness correlates if only two observed fitness correlate values are available (example: wild type reference and one knock-out mutation). If the relationship is indeed linear, adding more mutations will merely confirm this and increase precision. If the correlation is decidedly non-linear and the LFCH is falsified, the additional data can be put to more use than merely rejecting the current evolutionary systems biology model. Such data can be used to calibrate a map from the current computed fitness correlates to the observed fitness correlates. In situations like these, the adaptive landscapes of *level *5 are needed as a separate level (observed fitness correlates in the space of predicted fitness correlates; see above).

#### The power of linear extrapolations

Linear extrapolations are often particularly accurate, if the extrapolations are small. This is the mathematical basis behind much of calculus, as arbitrary functions can be composed of many small lines, where shorter lines lead to more precision. Applying this logic to the LFCH has several implications.

First, if a linear correlation can be demonstrated on both sides of a reference wild type point, then one can have a high confidence in computational results *within *that range, which implies that very small mutational effects can be predicted with a high accuracy. This is important, since these mutational effects have been very difficult (or impossible) to analyse with other methods so far.

Second, if the LFCH is rejected, one could still use the existing data points for interpolations that allow for arbitrary mappings with reasonable accuracy. Whatever interpolation or surface averaging function is used, smaller deviations than observed fitness correlate differences are again very likely to be relatively accurate.

Third, in the absence of any experimental calibration, continuous fitness correlates are still expected to behave approximately linearly in the immediate proximity of the wild type reference point that the model aims to represent. The selection coefficient of a mutation is defined relative to that of other alleles, so the absolute magnitude of fitness correlates is not needed for many evolutionary analyses. An unknown LFCH slope will result in the need to scale inferred selection coefficients by an unknown, but constant factor if one can assume that the LFCH holds. This allows biologically interesting statements about very small mutational effects as discussed below. To obtain precise selection coefficients, one only needs to scale results with a corrected slope. Without such a correction, one can still estimate the shape of the distribution of mutational effects, the fraction of advantageous versus deleterious changes, the frequency of compensatory changes and many observations about epistasis. Thus, many interesting questions can be addressed by assuming the LFCH in the absence of any calibration.

#### How to define computable fitness correlates

To define computable fitness correlates requires much biological intuition and can be considered an abstract form of art, like all modelling. However, a few guiding principles can greatly facilitate the process.

##### Focus on one fitness correlate at a time

The first important step towards their successful definition is to realise that computing fitness correlates is different from computing fitness. To calculate fitness, one needs to use a model of life history evolution that takes various fitness correlates as input [58,59,149,215-217]. Such a model may be complex or simple but it will always have clearly defined fitness correlates as input. Fitness correlates can be survival rates, reproductive output, growth rates, resource allocation strategies and/or many other properties that are frequently investigated in life history evolution. Thus, to successfully define a computable fitness correlate requires focussing on this particular fitness correlate and finding all lower-level processes, structures and functions that contribute to it (see below). While this is done for one particular fitness correlate all other fitness correlates can be ignored. After every single fitness correlate has been defined bottum up, they are combined in an overarching life-history evolution model that specifies fitness and potential trade-offs at the highest level.

##### Develop biological intuition

The next important step is to develop a good biological intuition for the system in question. This is less well defined than the first step, as it heavily depends on the specifics of that system. Many important hints can be obtained by talking to different experts that understand particular aspects of that system very well. Learning about the ecological functions of the system could be as instructive as the analysis of potentially interesting observations of phenotypic selection in the wild [152]. It is important to mentally put oneself in the system's place to develop an intuition for how it works. It might also help to imagine that one would have to engineer such a system in order to maximise the fitness correlate: Which properties would or would not be important? How are they related? How do they combine functionally to determine ultimate behaviour? Are there indirect effects that could be important? Current systems biology uses top-down and bottom-up approaches to arrive at complete and quantitative descriptions of particular systems of interest [5]. This requires the development of substantial amounts of intuition that is likely to be very helpful in the development of computable fitness correlates.

##### Use recurrent guiding questions

Recurrent patterns can be expected to emerge when repeatedly engaging in the definition of computable fitness correlates. Such patterns can be used to define guiding questions that might help to develop computable fitness correlates. Top-down and bottom-up approaches can be used to investigate all processes that might affect the answer to a particular question [5]. Such questions might include the following:

• How does this system impact the energy balance? Does a mutation lead to the consumption of more or less energy? Will a mutation help the acquisition of metabolic energy or food from the environment?

• How does this system impact the probability of survival? Is it important for fighting pathogens? Is survival endangered by a particular mutation that impairs the system? What happens to survival in various environments if the whole system fails? How frequently is this system critical for survival?

• How does this system impact mating success? Sexual selection can be a strong evolutionary force and various traits can acquire special importance by serving as signals during the selection of mates.

• Will this system make it easier to produce more offspring quicker?

• Does this system impact the reliability of other systems? The rate of errors during transcription and translation will affect the quality of proteins in a cell and the rate of DNA replication errors can change the probability of acquiring cancer.

There are many more questions that can be asked at a much more detailed level. It will be helpful to collect such questions and evaluate their usefulness to facilitate efforts to define fitness correlates.

##### Handle trade-offs

Many systems require more than one fitness correlate to accurately reflect their evolution. For example, all organisms rely on metabolic energy for survival, but if they accumulate too much of it, their probability of survival can be reduced by predators or obesity related problems. Such situations lead to trade-offs, where the evolutionary fitness optimum consists of a compromise between two important features that cannot both be optimal. There are two basic ways of handling such situations:

• **Limit the scope of the adaptive landscape**. The existence of trade-offs indicates that often there is a range of parameter space, where one or the other factor dominates. In the absence of an appropriate life history evolution model, one could limit the investigation to parameter combinations that do not require knowledge of the trade-off. In that case the side effects of changes in one fitness correlate on other fitness correlates can be ignored.

• **Build an appropriate life history model**. If such a model includes all fitness correlates that are affected by particular changes of the system, then much more general predictions of evolutionary optima become possible (see discussion of levels 6 + 7 above, which map fitness correlates to the fitness of an individual to the mean fitness of a population).

Ideally one will want to find the properties in a mechanistically understood molecular or tissue-level subsystem that limit the value of an observable fitness correlate. Using mechanistic insight, one will then define a set of equations and algorithms allowing the computation of a value that is expected to be linearly correlated with the observable fitness correlate. The goal is reached when computations and observations match as stated by the LFCH.

#### Examples of potential computable fitness correlates

Few computable fitness correlates have been defined so far, as the level of knowledge required for attempting such definitions has only become available recently. A well known example of a fitness correlate is the prediction of growth rates of microbes from total metabolic flux or total biomass production in flux balance analysis models of *E. coli *[108,12] and yeast [9,13-15,219]. The LFCH is supported in these systems, albeit with some limitations.

From work that combines experimental molecular biology and evolution come examples of molecular properties that affect energy metabolism and that map directly to relative growth rates as observed in a chemostat [95,112,113]. Indeed, it has been suggested for some time that measures of energy efficiency could be used as indexes of fitness to learn more about the organisation of biochemical networks [96,111,116].

Another example of a computable fitness correlate has been given recently in the work that preceded the more extensive description of evolutionary systems biology given here [196]. Using the example of a simple circadian clock it was demonstrated how a fitness correlate could be defined in order to capture the correct timing of recurrent gene expression if the latter is essential for growth. Briefly, in the system of interest an internal signal is identified that is used to switch on or off the genes that are optimal for a given external environment that changes regularly. The internal signal can be in sync with the external one or it can be completely out of sync or it can be dominated by randomness. Simulations of the system can be used to determine the fraction of all time, where the internal signal switches 'on' the genes (e.g. photosynthesis) that are optimal for the current state of the environment (e.g. day) [196]. The large impact of internal predictions of daily rhythms on various observable fitness correlates has been demonstrated experimentally [226,227]. It can be assumed that the absolute amount of time where genes are out of sync with the environment influences fitness directly. Thus there is scope for testing the LFCH in this system [196]; examples for insights into the distribution of mutational effects that can be obtained by assuming the LFCH in this system are discussed below.

Computable fitness correlates can be constructed for many other systems. For example, one could consider the probability that a signal transduction pathway triggers a defined response: using simulations it could be investigated how probable it is that a pathogen induced activation of a signalling molecule at the cell surface will result in the activation of a nuclear response that switches on the genes needed for fighting the pathogen (it is clear that survival is reduced if this probability is reduced). One could also estimate the amount of energy needed to produce proteins [228] and the speed and accuracy with which that happens [229-232]. Combining such estimates with simulations of ribosomes might help us to assess the impact of mutations in the translational machinery.

It is important to note that models, which attempt to define computable fitness correlates, do not have to be perfect in order to be useful. In fact, some important conclusions are not affected by the omission of many details. To appreciate the importance of this point one has to consider the extraordinary crude models of fitness that are often used with great success in evolutionary biology. Against that backdrop many simple inclusions of mechanistic knowledge in fitness models appear like major advances. For example, almost arbitrarily defined biochemical reaction networks have been used to investigate their evolution [233-236].

### Distribution of Mutational Effects (DME)

Since high-dimensional adaptive landscapes are very difficult to navigate and virtually impossible to visualise, researchers have been developing abstractions that provide a more accessible picture. One such abstraction is the Distribution of Mutational Effects (DME). Traditionally, two approaches have been used for estimating DMEs. The *experimental approach *accumulates mutations and directly estimates effects on an observable fitness correlate [51,68,237-241]. All experimental methods are labour intensive and unavoidable experimental errors make it impossible to observe small effects. This is a major limitation, since most mutational effects are expected to be below the threshold of detection [2,242]. Therefore, *population genetical methods *have become increasingly popular [2,1,66,243-245]. These methods combine an evolutionary model with observed DNA sequence data in order to estimate which DME explains the observed data best. They allow the detection of very small effective selection coefficients (on the order of 1/*N*_*e*_, where *N*_*e *_is the effective population size). However, they have almost no power to estimate very large effects and their results can strongly depend on the assumed model of evolution. Difficulties become even more pronounced, when these methods are used to infer the fraction of adaptive substitutions. Experimental and population genetical approaches have in common, that they estimate a generic distribution for a sample of sites in the genome. The resulting DME is descriptive and has no underlying mechanistic basis. Since the DME is of extraordinary importance, it would be desirable if a completely independent approach could be used to confirm findings. Evolutionary systems biology could provide such an approach, which was first described elsewhere [196]. This approach is based on current systems biology models with fitness correlates and does not suffer from the weakness of the other approaches. It can also be used to estimate DMEs that affect any emerging property and is not limited to fitness correlates. However, at its best it can only estimate the DME in a specific system, not in a representative genomic sample.

#### Observation of DMEs *in silico*

To observe a DME for a well-defined biological system with a corresponding computable model one can use the current wildtype as a point of reference and then compute the fitness effects (or other effects) of many random mutations, using the following steps:

1. Choose an interesting wildtype parameter set as starting point.

2. Choose a realistic distribution of mutational effects on the kinetic parameters to model the effects of changes in DNA sequences on kinetic parameters (see discussion below).

3. Scale the frequencies of mutations that affect the various kinetic parameters according to the size of their respective mutational targets and their corresponding mutation rates. Bigger proteins usually mutate more often than smaller ones.

4. Do many one-step random perturbations of the wildtype and compute the mean fitness (or other property) for each of them to as many decimal digits as you need to measure the smallest selection coefficients (or other effects) that you want to predict.

5. Summarise the differences to the reference genotype as a DME. Since a logscale is best to visualise the expected majority of small effects, it may be preferable to use two log-scaled distributions, one for deleterious mutations and a separate one for advantageous mutations (see [196] for a plot that was especially designed for visualising the DME).

#### The quality of *in silico *DMEs

The quality of DMEs inferred by this approach depends on the quality of the underlying systems biology model, the fitness correlates, their calibration and the assumed distributions of kinetic parameters. If high quality fitness correlate calibrations and realistic distributions of kinetic parameters are available together with estimates of *N*_*e*_, the effective population size of the species that carries this system, then the fraction of effectively neutral changes can be computed approximately by lumping together all mutational effects with a selection coefficient *s *< 1/*N*_*e*_.

To estimate the overall fraction of absolutely beneficial mutations no calibration is necessary. It suffices to determine how many mutational effects increase or decrease the fitness correlate. The overall type of this distribution should also be obtainable without accurate scaling, allowing tests of the expectation that advantageous mutational effects are distributed exponentially [2,142,240]. However, to determine the fraction of effectively beneficial mutations, a proper scaling becomes important again, since such mutations have to be distinguished from effectively neutral mutations. Again, effectively neutral and advantageous mutations are approximately separated at *s *≈ 1/*N*_*e*_.

Estimating a precise DME requires knowledge of the first two *levels *of adaptive landscapes described above (mapping from genotype to molecular structure to molecular function). In other words, it is important to know how DNA changes translate into changed kinetic properties for the macromolecules in the system. Several solutions to this problem are possible. Ideally, one might want to introduce the random changes at the level of DNA sequences and use a reliable molecular ***ab initio *prediction **system to determine the resulting changes in kinetics that are then used as input for the corresponding systems biology models. Unfortunately, this overstretches the capabilities of current *ab initio *modelling approaches [164]. The two next best solutions have been described above in more detail while discussing the first two *levels *of adaptive landscapes. One solution is to use **comparative modelling **of structures based on changed sequences and then comparative modelling of kinetic properties based on changed structures. These methods work particularly well for small changes and thus appear suitable for investigating DMEs. However, currently no ready-to-use pipeline exists that allows non-specialists to go the whole way from DNA sequences to kinetic rates for all proteins that are known well enough. The other next best solution is to employ **random mutagenesis **experiments to measure the distributions of kinetic changes that are caused by random DNA changes in the corresponding genes. Technological advances might help to increase the accuracy of measurements enough to capture all important changes for DME predictions and to reduce the corresponding work enough to allow regular use of such technology for assessing new kinetic properties in new proteins. However, at the moment such random mutagenesis experiments are laborious and functional assays for most genes are probably not accurate enough to capture the overwhelming majority of very small effects that are expected in DMEs based on population genetic inferences [2,93]. The third best solution is to use **specific reasonable evidence-based assumed distributions **for how kinetic parameters change with DNA sequence changes. This approach uses general observations from comparative modelling and random mutagenesis experiments in generally similar macromolecules to propose reasonable estimates of the expected distribution, even if no specific data are available for the specific genes under consideration. The emergence of universal patterns of some effects in proteins support this approach [172,173]. However, reducing the rigour applied in the construction of such assumed distributions puts one on a slippery slope towards **arbitrary assumptions**. As this solution is least desirable in terms of quality and most desirable in terms of ease of implementation, one might ask if such a large unknown does not necessarily invalidate all other simulation efforts at higher levels. While there is clearly the potential for this to happen, a sensible simulation strategy can nevertheless learn valuable insights from employing such an approach:

• **Three point estimates**. One could use an approximate minimum, most likely and maximum value for the kinetic parameter in question to assess its expected contribution to the computed fitness correlate. The corresponding computations are easy to do and should quickly indicate, whether the parameter in question is stiff or sloppy (see Box on modelling below). The former highlights the need for more in-depth analysis that could justify experiments, while the latter might suggest that the role of this parameter is too small to warrant further attention.

• **Varying distributions**. If preliminary calculations indicate that a kinetic parameter is of some importance, one can assume several distributions of this parameter that are chosen to be as different as possible, but still compatible with the limited data available. Using such an ensemble of distributions of different types and with different location and shape parameters, one can ask how high-level emergent properties are influenced by assumptions about low-level distributions of kinetic rates. A possible result could be that many features of low-level distributions have only little influence on high-level distributions. Such parameter sensitivity analyses can guide experiments to investigate those properties of low-level distributions that make a difference *in silico*.

#### DME nomenclature and DME plots

As evident from the text above, systems biology analyses of mutational effects generate many different DMEs at various *levels *of the adaptive landscape. They differ in the *mutational *input they assume (equivalent to the *plane *of a landscape) and in the output *effects *they produce (equivalent to the *height *of a landscape). To specify DMEs precisely and concisely a new nomenclature was developed [196]. Since all biologically interesting properties of DMEs are notoriously difficult to visualise in one plot, a special new type of histogram was designed to provide a quick visual overview over DMEs [196].

#### Exemplary results

To illustrate the power of this new approach, a systems biology model of a very simple circadian clock mechanism has been analysed with the help of stochastic simulations [196]. This model assumes an almost arbitrary distribution of kinetic effects of DNA changes to bridge *levels *1–2 of the adaptive landscape. It then simulates a very simple systems biology model at *level *3 and defines a computable fitness correlate at *level *4. In a further gross simplification the computable fitness correlate is assumed to be identical with the actual fitness of an individual (jumping *levels *5–6). Here are the key observations from that analysis:

• **Null-Hypothesis**. In some situations it appeared that changes in the low-level distribution of kinetic rates were closely mirrored by changes in the high-level distribution of an emergent property of the system. This appears to be trivial enough to serve as a null-hypothesis against which 'canalisation' could be detected (see section on robustness below).

• **Inversions**. In some situations a decrease or increase of the low-level rate led to the corresponding opposite effect on the emergent property. It will be interesting to see how frequently such inversions can be found in more extensive studies.

• **DMEs are context dependent**. Epistasis was expected in this system. However, it is interesting that this work opens a new approach towards quantifying epistasis. With the extensive possibilities for manipulating such simulation models, epistasis research might be able to address new questions.

• **Changing a good clock usually degrades it**. Again, this was expected, but it is reassuring to recover such common sense results from simulations. Future work will be able to quantify much more precisely how many out of all possible low-level parameter changes are expected to be harmful at higher levels.

• **Changing a bad clock can improve it**. It was possible to detect advantageous mutations in the limited parameter space searches that were conducted. It was particularly interesting to find a parameter combination, where both increase and decrease of a particular kinetic parameter led to fitness correlate increases. Further work will have to investigate how frequent such situations are.

Many questions can be asked about the simple simulation model employed in this study (the clock model has no entrainment and ignores most of the interactions that circadian clock research has uncovered in recent years) and the usefulness of the conclusions drawn from it. All these questions can be addressed by building more realistic models; the main purpose of this pilot study was to demonstrate the type of data that this approach can generate [196]. Future work can use the same principles to arrive at much more precise and interesting results.

#### What can be expected from such analyses?

While any single analysis will not be very insightful from a general point of view, there are two big questions one would like to ask of a reasonably sized sample of such analyses: First, how different are the DMEs for different systems? A system in this context stands for any molecular systems biological model – no matter how small – that allows the computation of a fitness correlate. It is conceivable that each system has its own very peculiar DME and cannot be compared to any other known system. However, it is equally conceivable that the general properties of complex systems somewhat smooth out the differences, which would imply that most DMEs look rather similar.

**Figure 4:**
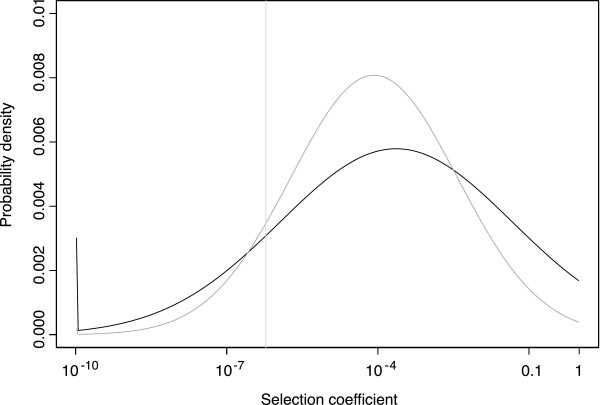
**A potential distribution of deleterious mutational effects**. Selection coefficients range from effectively neutral on the left up to lethal on the far right. The vertical line denotes the border between effective neutrality and effective selection. Both distributions assume a lognormal law, the light grey distribution also assumes a separate class of completely neutral mutations with a frequency of 2.5%. Estimates applied an evolutionary model that included mutation, selection, genetic drift and backmutations [1,55] to data from *Drosophila pseudoobscura *and *D. miranda*. For more details, see [93]. All selection coefficients beyond 1 ('super-lethals') denote an abstract notion of structural damage to the organism. Such damage cannot be more than lethal and is thus represented as lethal in evolutionary models.

Second, if such general properties exist, is it possible to find a theoretical justification for the expected distribution? A recent comparison of various types of distributions of deleterious mutational effects has found that the lognormal distribution explained the data best in that example [93]. If the extent of a reduction in fitness caused by a deleterious mutation is a multiplicative function of the damage that it causes at several independent functional levels, then one expects a lognormal distribution of mutational effects [93]. It will be interesting to see if a lognormal distribution, as in Figure 4, turns out to be a reasonable null-model for the distribution of mutational effects on fitness in the long term.

In any case, the DME is of such fundamental importance for robustness [246], and so difficult to estimate, that multiple approaches are needed to develop confidence in any particular result [2]. The strength of the approach proposed here is that it opens up access to very small mutational effects without the need for assuming or inferring a particular evolutionary model. Thus it can be seen as a third principal approach besides direct experimental measurements and DNA sequence based inferences that assume evolutionary models [196].

### Robustness, canalisation and capacitance

Canalisation reduces the sensitivity of a phenotype to changes in the underlying factors that determine its expression [247,248]. It is important for understanding robustness which is of interest to current systems biology [246] and current systems biology models have been used successfully to investigate it [249,250,199-201]. Canalisation was introduced by Waddington to explain the robustness of phenotypes that he observed in experiments [251] and researchers have struggled to provide a precise basis for quantifying it [247,252]. Recent work has started to uncover some of its underlying molecular basis (e.g. [253]). The opposite of canalisation has been termed 'capacitance' to highlight the adaptive possibilities that can come with the expression of new heritable phenotypic variation [250,126,253,254]. The consequences of robustness, canalisation and capacitance can be very obvious in many developmental pathways due to their effect on morphology. Thus these concepts play a major role in 'evo-devo' that combines evolutionary biology and developmental biology [124-126,199-211]. The concept of robustness is also pivotal in the study of biochemical reaction networks [246,255-260].

The analyses of DMEs above open up a new approach towards measuring canalisation rigorously for a pair of DMEs. One of these DMEs needs to be a low-level DME like the distribution of mutational effects on the kinetic properties of a given enzyme. The other DME needs to quantify an emergent property of the system like the production rate of biomass. The different scales of low-level and high-level properties are mapped to the unit-less scales of DMEs that record relative deviations from the wild type on their *x*-axis. Thus, the null-hypothesis is true, if for all possible values of *x*

**Figure 5:**
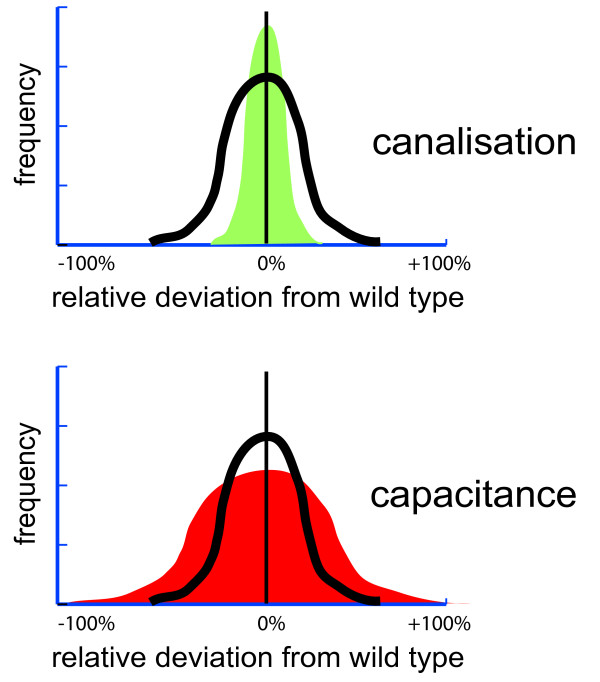
**Canalisation and capacitance measure robustness**. Let's assume a low-level property such as the kinetic rate of an enzyme has a certain distribution of mutational effects (black line) and influences a high-level property such as a fitness correlate (filled area). If the variance of the high-level property is smaller than that of the low-level property, then the high-level property can be said to experience 'canalisation'. Conversely, the high-level property experiences 'capacitance' if its variance is larger. To make these two properties comparable, their values on the *x*-axis are both plotted as relative deviations [(*x*_*mutant *_- *x*_*wild*_)/*x*_*wild*_].

*x*_*DME *_= (*x*_*m*,*low *_- *x*_*wt*,*low*_) /* x*_*wt*,*low *_= (*x*_*m*,*hi *_- *x*_*wt*,*hi*_) /* x*_*wt*,*hi*_

where the indices *m *and *wt*, *low *and *hi *denote mutant and wild type values for low-level and high-level properties, respectively. Then canalisation is defined as occurring when the variance of the lower level DME is larger than that of the higher level DME (Figure 5). Correspondingly, one might observe capacitance as the opposite of canalisation, when the variance of the lower-level DME is smaller than that of the higher-level DME (Figure 5). This definition applies equally if *x *is plotted on linear or logarithmic axes, as long as the same transformation is applied to both DMEs (logarithms facilitate visualising very small mutational effects; for more details on how to produce DME plots, see [196]). Such analyses can explore the question whether high-level DMEs depend more on the intermolecular interactions captured in complex systems biology models or on the low-level intramolecular interactions within proteins that determine reaction rates. While both are expected to contribute, a more precise answer is important for current systems biology modelling in general. Canalisation and capacitance are caused by epistatic interactions.

### Epistasis

Another abstraction of the adaptive landscape is the distribution of epistatic effects. Epistasis is defined as any deviation from an independent combination of mutational effects, which under a multiplicative fitness model is obtained by simply multiplying the fitness values of both mutants. Evolutionary genetics has explored many of the enormous consequences of particular types of epistasis [90,92,261,262,248,247,91]. However, there is much uncertainty when it comes to determine which type of epistatic interactions occurs how often. Studies so far have consistently demonstrated that synergistic and antagonistic epistasis are fairly common in nature and while they might almost cancel each other out on average, they show significant variance around their mean [94,16,262,263,143]. Less is known about the frequency of sign epistasis, which decreases the fitness of intermediate mutants below that of the wildtype, even though the fitness of the final mutant is above that of the wildtype [91,92]. Despite the importance of epistasis in evolution, models that explore evolution in the presence of epistasis use rather simple models of epistasis and often allow only for one constant type of epistasis. Since epistasis is caused by the underlying molecular interaction networks, the use of fitness correlates as defined above can help explore it. Indeed some early work has used metabolic control theory to investigate epistasis [262] and the origins of dominance [114]. Recent work has suggested that a synthesis of current systems biology models and quantitative genetics methods can successfully investigate epistasis [264,265].

To examine the distribution of epistatic effects in the simplest case one may consider just two mutational steps:

**1. Reference**. Compute the fitness of the current wildtype as a reference (*W*). Generally compute mutational effects as if estimating a DME, so the same caveats apply.

**2. Independent estimates**. As in analyses of the DME, these always start with the wildtype. Add each random mutation separately to the wildtype and compute the resulting fitness. Thus, the two mutations A and B will result in the fitness values *W*_*A *_and *W*_*B*_, respectively.

**3. Combined estimate**. Starting with the wildtype, add all mutations from the previous step at once and then compute their combined effect on fitness. This results in one single fitness value, *W*_*AB *_in our example.

**4. Compare**. In the absence of epistasis the product of all independent fitness values equals the combined fitness of all mutations in one genotype (if fitness is multiplicative). The difference between these two indicates the type and size of the epistatic effect.

**5. Distribution**. To obtain a distribution of epistatic effects, repeat (2) – (4) for many random perturbations of the system. At the same time consider quantifying sign epistasis [91].

Repeating such an analysis for several different fixed starting points gives a crude high-level overview of epistasis for the model under investigation. The procedure above outlines only the simplest case of '2-step epistasis'. The resulting distribution will strongly depend on the number of mutational steps analysed, so *n *different mutations need to be analysed for one measurement of epistasis in order to understand *n-step epistasis*. To get a thorough understanding of 'general' epistasis in a system, one needs to analyse the distribution of epistatic effects for a wide range of different *n*.

A distribution of 2-step epistatic effects has been obtained *in silico *by using flux balance analysis in yeast [16,266]. The approach above extends *in silico *methods to general current systems biology models that often cannot be analysed with flux balance analysis. As with the DME, the main strength of this framework is in facilitating many independent observations that help searching for general patterns (or documenting their absence). Analysing *n*-step epistasis for large *n *can also help investigate the frequency of compensatory mutations at the molecular level, a question that has recently been addressed experimentally [76-78].

### Testing evolutionary hypotheses

Hypothesis testing in evolutionary biology has become increasingly important and sophisticated [267,105]. Many phylogenetic hypotheses completely ignore selection and treat all DNA sequences as neutral, although they implicitly consider the effects of selection by allowing for different rates at which new mutations are substituted along lines of descent. This approach has been very successful in testing various hypotheses [42,267-270].

In contrast, many population genetic hypotheses explicitly test for selection. Using selective neutrality as a null-hypothesis, population genetic tests can detect positive or negative selection that causes deviations from neutral patterns of DNA sequence evolution [105,42,1,93,60,271,272,244]. Such work can highlight sites in the genome that are under a given type of selection. In some cases the results may be specific enough to inspire mechanistic explanations for the molecular causes behind the observations. Indeed, genomic analyses have been searching for 'candidate genes' of potential medical interest [67,62].

However, current population genetic studies have no formal power to distinguish between different mechanistic explanations for *why *selection operates where it does if these explanations suggest the same DNA sequence patterns. Such questions could be answered with some rigor if fully developed evolutionary systems biology models were available as null-hypotheses. The ability to construct such models is closely linked to the ability to predict the likely courses of evolution out of all potentially conceivable courses of evolution (e.g. [91,157]) and is related to the functional synthesis of molecular biology experiments and evolutionary theory that was used to reconstruct ancient adaptive events [95,112,113].

The ultimate goal of evolutionary systems biology is to quantitatively test evolutionary hypotheses that are fully mechanistic, predict all phenotypes of interest *ab initio *from their respective genotypes and that are ecologically realistic (Figure 1). This ambitious research programme critically depends on the various more specific research programmes described above that predict phenotypes from genotypes with the help of computable fitness correlates. These predictions need to be incorporated into realistic ecological and population genetical models that describe how selection and the various other evolutionary forces affect a population of individuals. Provided enough computing power is available, evolution under the resulting model could be observed in individual-based simulations by applying the evolutionary forces of mutation, selection, genetic drift, recombination and migration to a population that moves on the adaptive landscape. Tracking the evolution of such a population *in silico *and comparing these results to observations could provide a unique capability to test complex evolutionary hypotheses (Figure 1). For many questions the evolving population will consist of individuals in an ecosystem. However it can also consist of cells in a body, a perspective that is pivotal for understanding the origins and progression of cancer [192]. Evolutionary systems biology simulations could point out gaps in our current understanding of a system (e.g. see [273]) and thus motivate further work towards the construction of hypotheses that are both quantitative and free of conflicts.

Developing evolutionary systems biology up to the point where such analyses become commonplace is a 'grand challenge' and will take a long time if it is possible at all. We can expect to gain many new insights by working towards this goal, even if such analyses are too complicated to become commonplace. Such work will contribute towards unifying biology (Figure 1) and will need to integrate various approaches towards understanding biology (Table 3).

**Table 3:** Approaches towards understanding biology.

**latin**	**meaning**	**strength**	**weakness**	**analogy^1^**
*in ratio*	analytic model	well understood, precise predictions or approximations; can falsify intuitions and hint at simulation errors; can explain data if mechanistic	limited to simple models by mathematical tractability	hard, dry bone

*in silico*	simulations of more realistic models	can be very realistic; can use more observations than analytic models to make better predictions; can falsify approximations and intuitions; can explain data if mechanistic	sometimes too hard to understand; computing can be costly; some heuristic models can predict data without explaining	flesh

*in vitro*	experiment without anything alive	precise molecular observation and manipulation possibilities; can falsify models	can be expensive; extrapolation to in vivo is not always possible; complexity limits	food to eat

*in vivo*	laboratory experiment with living cells	controlled environment allows specific manipulations; can falsify models	relevance for natural settings not always clear; limited mechanistic understanding	water to drink

*in natura*	observation of organisms in their natural setting	get information on actual natural processes; can falsify models	either only historic or usually limited by ~3 year funding periods; limited mechanistic understanding	air to breathe

*in tuitio*	ask good questions	very cheap and fast; all ideas start here	is no scientific proof in itself	spirit with good ideas

^1 ^As analogy, think of a biologist made of flesh and blood. Just bones and flesh are dead unless they breathe air, drink water and eat food. This illustrates that good theoretical models need to be designed to incorporate experimental data in order to 'come alive'. A good intuition is needed to develop such models. Heuristic models can be very good in predicting observations, but true understanding grows only when models reflect true causalities.

## Discussion

There are historical precedents for successful interactions between evolutionary genetics and systems biology as evidenced by the interactions and interests of some of the founding fathers of the respective fields. Kacser used metabolic control theory to explain the molecular basis of dominance [114]. In doing so he supported Wright's hypothesis on the same topic [185] and contributed to a long debate in evolutionary genetics [274-277]. Metabolic control theory was also used to make predictions about the expected intensity of selection on enzymes with different control coefficients [183,184,187,115] and about epistasis [262]. Others have long suggested a development similar to evolutionary systems biology, namely bringing together the analysis of bioenergetics and evolution [95,96,111-113,116]. Interactions are by no means a one-way street. J.B.S. Haldane, who is best known as an evolutionary biologist, used the quantitative skills he developed for population genetical analyses to make a fundamental contribution to systems biology by introducing the quasi-equilibrium approximation to Michaelis-Menten kinetics [278], still widely used in current systems biology. Likewise, current systems biology can benefit from expertise in the quantitative analysis of complex systems that has been developed in evolutionary biology.

This study proposes a multilayered mechanistic framework for evolutionary systems biology (short EvoSysBio) that centres on fitness, the adaptive landscape and the quantitative modelling of evolutionary processes. However, other approaches to EvoSysBio are possible too. For example, comparative EvoSysBio can analyse how phenomenological descriptions of systems like gene networks differ across species [279,280]. Comparative EvoSysBio can help identify functionally important differences between species. It flows naturally from the wide availability of systems biology data sets for many species. EvoSysBio can also be approached in a 'target-oriented' way without the principled framework described above. One could define target-oriented EvoSysBio as combining at least one current systems biology approach with at least one evolutionary genetics approach to facilitate the understanding of a particular system. For example, one can study correlations between the various systems biological and evolutionary properties of genes [280]. Also, the large interest of current systems biology in cancer and the evolutionary nature of cancer naturally inspire such EvoSysBio work [281]. Network-oriented EvoSysBio can be considered as target-oriented EvoSysBio aiming to understand the evolution of generic features of biochemical networks like robustness [281,255-260,282]. All these approaches to EvoSysBio can produce valuable insights without the framework presented above. Many of these insights are likely to contribute towards building the multilayered mechanistic EvoSysBio models proposed above. The goal of constructing such models is expected to inspire the generation of a wide range of quantitative hypotheses and critically depends on a diverse body of detailed work in many fields. Such work has not been labelled 'EvoSysBio' (and does not need to be).

Readers with mathematical skills will have missed the formal definition of many important concepts in the overview presented here. This is in part due to space limitations that prohibit a proper review of concepts that have been developed elsewhere (see references cited). Further, the aim of this article is to provide a motivational overview of the new field of EvoSysBio in order to inspire the development of corresponding formalisms. Such formal definitions will facilitate the proposal and rigorous testing of many new hypotheses. The success of EvoSysBio critically depends on progress towards properly quantifying the concepts presented above. This will be strongly influenced by answers to the following critical questions.

### Critical questions

The limited evidence that already exists makes it easy to guess preliminary answers to the following critical questions one may ask about EvoSysBio. Obtaining more reliable answers depends on the investigation of a multitude of systems, amounting to a major research program.

• **What proportion of current systems biology models allow the definition of meaningful fitness correlates that possess enough accuracy to be useful and that are still computationally tractable?**

It is clear that some such systems can be defined, but it is not clear how difficult this will be for 'typical' biological systems of interest. Thus it will also be interesting to describe systems where no meaningful fitness correlates could be found. It will be interesting to see if it can be formally defined how 'useful' a proposed fitness correlate is or whether this will remain in the domain of biological intuition.

• **What proportion of the results of evolutionary systems biology analyses are similar to one another?**

If common patterns emerge, it might become much easier to analyse more systems. If all results are highly system specific, this will help avoid unwarranted generalisations.

• **What proportion of the distribution of mutational effects on fitness is determined at which level?**

Do intra-protein interactions that only affect kinetic parameters contribute more than intra-cellular reaction network interactions or still higher levels of functionality that affect emergent properties more directly? In other words, where does most of the canalisation happen? If most of the variability is caused by intra-molecular interactions, then the corresponding data is vital for the overall success of current systems biology models in this context. How important is a detailed understanding of structure-function relationships within proteins for understanding the robustness of molecular systems biological models?

• **What proportion of all model input parameters can be determined with enough accuracy for the analyses proposed here?**

A typical critique of 'model everything' approaches is that there are too many parameters that one would need to know for such models to be of value. Analyses have shown that in most models not all parameters are of equal importance [194]. Thus it might not matter, if some parameters remain poorly defined, as long as one can develop methods to demonstrate that these parameters are not pivotal for the models of interest. Will it be possible to determine every important parameter with sufficient accuracy?

• **Which approach can accurately predict most molecular kinetic parameter changes that are caused by DNA sequence changes?**

Can experimental approaches attain the level of precision required for evolutionary analyses or do we have to rely on computers? Is precise *ab initio *modelling possible on commodity hardware or does this always require super computers (if possible at all)? Can experimental random mutagenesis approaches be faster in determining low-level DMEs than *ab initio *modelling approaches? The worth of each method for practical use is a trade-off between the cost, speed and accuracy of prediction. It is unclear whether high-throughput experiments or high-performance computers or very clever algorithms will dominate eventually, as the development of all three approaches progresses very fast.

• **What proportion of predicted distributions of mutational effects or distributions of epistatic effects can be confirmed in the laboratory?**

In principle, one should be able to devise corresponding mutagenesis or evolution experiments, but it is unclear how much power they usually have for testing *in silico *models. Experiments are integral for calibrating fitness correlates but they should also play a role as measures of quality control for completely integrated evolutionary systems biology models. Synthetic biology can also contribute towards testing evolutionary systems biology models.

Will the excitement in current systems biology survive the forces that ended a similar wave of excitement about modelling in ecological systems biology a few decades ago [40,41]? The answer is likely to depend on the quantitative rigour of the models and the quality of their links to observed biological data (see Table 3). An evolutionary perspective might contribute towards such quality. While molecular biologists do not need to become evolutionary geneticists and vice versa, some understanding of both fields is helpful for contributing towards the synthesis presented here.

### Benefits for current systems biology

Evolutionary perspectives can contribute much to current systems biology, as well as to many related agricultural [283] and medical [192] questions. Here are some examples:

• **Robustness **needs to be understood for improving drug-design [284]. Distributions of mutational effects need to be analysed in order to minimise medical side effects, as patients will carry the corresponding mutations. This is particularly crucial for drugs that are used on the long term.

• **Diseases**. Understanding cancer drives much interest in current systems biology. It is less appreciated than probably necessary that cancer is, by its very nature, an evolutionary problem: A population of mutating cells gains selective growth advantages in an environment and starts to evolve into a meta-population by building metastases. To understand cancer means to understand the evolution of these populations of cells. Thus many concepts that are of importance in population genetics are also pivotal for understanding cancer, including the distribution of mutational effects, epistasis and robustness of the various genes that are involved in producing cancer [192,285,286].

Improved mechanistic models of cancer could have practical implications too. For example, current predictions of life expectancy are usually based on regression analyses of data that shows how long patients survived if they shared particular properties like specific mutations [287,288]. In the long term it might be possible to construct mechanistic models of the corresponding signal transduction pathways and other processes that might add a more rigorous basis and possibly more precision to such estimates. Recent work points into this direction [289]. Mechanistic evolutionary models have also helped to understand other diseases (e.g. the Apert syndrome [290]).

• **Resistance**. The evolution of antibiotic resistance is one of the big problems of our time and if new drugs do not come with instructions on how to slow down resistance evolution, their effectiveness can be rather short lived [291-293]. Detailed evolutionary systems biology models could predict resistance evolution *in silico *and thus help to develop approaches to reduce resistance evolution. The same holds for other pathogens. For example, the HIV research community relies on an understanding of HIV evolution for developing therapies [294].

• **Agriculture**. A thorough understanding of long-term evolution is essential for a sustainable use of natural resources [295]. Informed decisions are needed about how to use the new crops with increased yields that can be generated by plant systems biology [36,283,296-298].

• **Synthetic biology**. Insights into the distributions of mutational effects are vital for understanding robustness and thus for both, the genetic engineering of synthetic biological systems and the genetic modification of existing ones. The prominence of engineering principles in synthetic biology [299-301] highlights the importance of understanding all sources of variability in the system. Each instance of a responsible release of these organisms into the wild requires thorough ecological analyses of the synthetic organisms' evolutionary potential to avoid unnecessary damage to existing ecosystems.

• **Population genetics**. Some current systems biology models can be enriched by including data from population genetic surveys of single nucleotide polymorphisms [302].

An evolutionary perspective can inspire new questions about current systems biology models by calling for investigations of distributions of mutational effects, epistatic effects and their long-term consequences. This is especially important, if the molecular systems under investigation exist in large populations for frequent long-term use *in natura*, as this allows small changes to add up to large consequences. The approach for estimating mutational effects presented here promises to be much more sensitive than current experimental methods and may thus increase our ability to predict evolution on longer timescales.

## Conclusion

**Figure 6:**
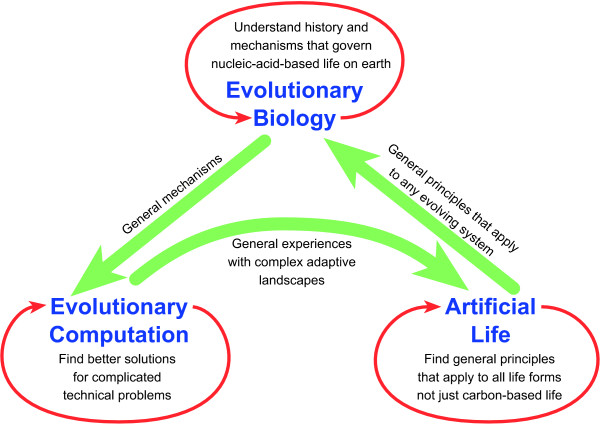
**The three fields that study evolution**. The red arrows encircle a goal central to each field. The green arrows denote major flows of inspiration and results. Each field has its own research agendas, conferences and journals.

The new framework presented here facilitates exploring general characteristics of living systems by combining current systems biology and evolutionary theory in order to address some of the most difficult problems in biology, including the distribution of mutational effects, robustness and the distribution of epistatic effects. These concepts are different ways of making statements about the adaptive landscape that governs the evolution of life. The methods suggested here will facilitate limited excursions into the adaptive landscape of particular molecular systems. These excursions will provide results that live between two extremes: (i) either each system is completely different and generalisations are virtually useless or (ii) the general complex nature of most systems will lead to fairly stable general properties that are easy to predict once the basic patterns are understood. An absence of experiments to calibrate fitness correlates limits the precision and hence applicability of results gathered by the proposed framework. In that case answers will only be rough and qualitative. Given the crudeness of many current models of fitness effects in evolutionary biology, this will nevertheless be a significant step forward, especially if many such rough models are built and common features start to emerge. Such experience will facilitate a deeper understanding of the adaptive landscape in evolutionary biology and may motivate exchanges with the other two fields that investigate adaptive landscapes: evolutionary computation, which investigates the adaptive landscapes of complex engineering problems and artificial life, which investigates general properties of life at the most abstract level (Figure 6). The advances in the field of artificial life suggest that fundamental insights might be gained from such exchanges [303-305]. The feasibility of exploring high-dimensional functional landscapes with the help of molecular systems biological models has been demonstrated by a computational study that investigated the sloppiness of parameter sensitivities. This study compared 17 detailed models and suggests that universal principles might exist [194]. The effort to make such analyses useful for evolutionary questions should be manageable and pay rich dividends.

Evolutionary systems biology has already been described as a nascent field, albeit in a context that either compares phenomenological descriptions of systems across species or that dissects correlations between multiple genome-related variables [279,280]. Emerging work at the interface between functional molecular biology, genomics, systems biology and evolution (e.g. [108,306,13,87,16,302,307,107,196,95,96,111-113,256,279,280]) is bound to lead to the growth of evolutionary systems biology approaches. Quantitative rigor as described in Appendix 1 will be pivotal for the success of such work. *Nothing in biology makes sense except properly quantified in the light of evolution*.

## Appendix 1: The art of modelling

The goal of modelling in biology is simple: describe an abstraction of reality that predicts natural processes, is mechanistically understood and remains as simple as possible. Since reality is complex, scientists often engage in a quest for models that increase in complexity at an astonishing rate, sometimes at the expense of clarity. While this causes some to argue for simpler, more reductionist models, others emphasise complexity to approximate reality more closely [308]. As much of this debate is based on personal preferences, one could take a more pragmatic approach, assuming that

• All models are wrong, but some are useful [309].

• Sometimes simpler models are better, unless statistical evidence demonstrates a significant increase in predictive power for a more complex model.

• Sometimes more complex models are better, unless it can be argued convincingly that all additional complexity does not impact the model behaviour significantly.

• Useful models have to be falsifiable.

• Errors need to be managed, for example, by starting simple and then adding complexity after the simple model has been understood. Starting complex can make errors difficult to find due to a lack of understanding.

• Models are there to do a job; time for model construction has to be limited if time should be left for analysing models in the real world.

This mix of Occam's razor, Popper's philosophy [310], practical advice and statistical theory [311,312] is a powerful tool for understanding our world and has been particularly successful in molecular biology and evolutionary theory (e.g. [42]). The importance of the simplicity of models is hotly debated, as some fear that adding parameters will obscure the core effects that are being analysed. Others fear that an artificial restriction of complexity will probably lead to the omission of key parameters that have a huge influence on the overall prediction errors. This fuels the desire in current systems biology to build comprehensive models that faithfully map the whole system and are independent of the questions that one might ask about such models. This approach is fundamentally different from the reductionistic perspective, as these complex models contain all the logic to 'simplify themselves' if only a simple question is being asked; in contrast to that, in the reductionistic approach the researcher performs the task of simplification by selecting what to include in a simple model. It is difficult to decide in general, whether researchers or automatic formalisms make fewer errors in simplifying complex models; the answer strongly depends on the formalisms and the system studied. It is not difficult to predict that human researchers working on any non-trivial modelling project will introduce errors that can matter scientifically and are challenging do detect. Having more than one strategy for debugging is extraordinarily helpful here. In the context of the debate on reductionism vs. holism, it is important to note the advantage of starting model construction at the simple end and progressing towards larger complexities, as the simpler models are understood.

As complex models are notorious for their computational complexity, a compromise may be helpful. One can build the more complex models and explore in computational parameter sensitivity analyses which processes exert how much influence on the system. Thus it becomes possible to identify parts of the model that are indeed unimportant for a particular problem, while being sure that all known processes are being considered. In a second step the unimportant parts can be omitted to save computing time.

Such an exploration of complex models is made feasible by computational advances. A steady stream of new research has driven knowledge far beyond what must have once appeared as science fiction. In molecular biology this has led to the recent emergence of current systems biology [29], which aims at integrating data about molecular processes inside organisms, often using approaches inspired by engineering [212,213]. Evolutionary theory is equally successful and has become an essential tool for analysing genome sequences [313,61,62]. Both branches of biology owe much of their success to the various complementary ways of understanding biology listed in Table 3. The recent increases of computing power and the need for more complex models have also led to a rise in computer simulations that aim at bridging the gap between the simplicity of tractable analytical models and the complexity of reality.

**Figure 7:**
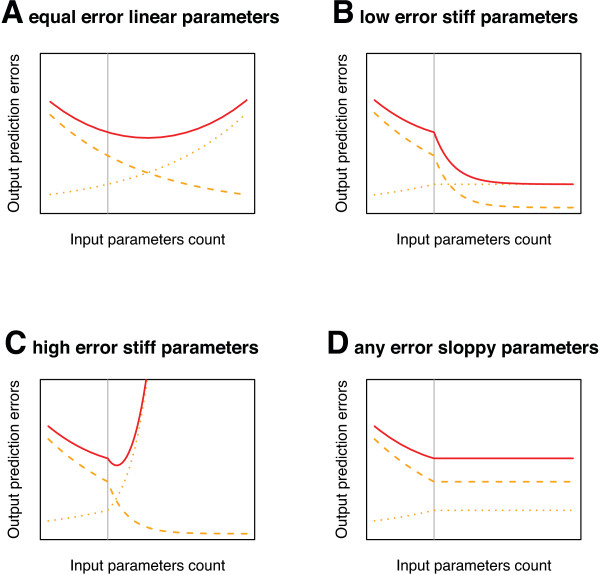
**The trade-off between systematic and random errors in modelling**. The number of input parameters is an indicator of model complexity and the sum of output prediction errors denotes the accuracy of the model with a particular parameter combination. The dotted line is the total random error introduced by badly estimated input parameter values, the dashed line is the total systematic error that comes from using a poor model with too few input parameters and the solid line is the combined overall error. See the text for more explanations. Note that sloppy parameters can become stiff and vice versa if the underlying model logic is changed to accommodate new parameters. All parameters left of the vertical grey line behave in the same simple linear way as depicted in (A).

Traditionally, biologists were only aware of two approaches to understanding biology: experiment and theory, where many theoreticians greeted with suspicion every model that was not completely analytically understood. Table 3 suggests a more differentiated picture that includes mechanistic computer simulations of various degrees of complexity as an equally valid approach to knowledge. It is important to strike the right balance between reductionism and realism [308] while using the right tools and asking the right questions – which makes modelling a form of abstract art. To strike the right balance it may be helpful to consider Figure 7 that depicts various possible trade-offs between the systematic error caused by using a simplified model and the random error caused by using imprecise parameters [314,315]. If all errors are equal and combine linearly (Figure 7A, [315]), there will be an optimal model complexity that is related to the 'Medawar zone' that describes the payoff and complexity of scientific problems ([316,317]. However, in non-linear systems there will be 'stiff' and 'sloppy' parameters that exert large and small influences on the prediction errors of output parameters, respectively [194]. If some parameters are stiff and well known (Figure 7B), it will be advantageous to include them. If the same stiff parameters are poorly known, one will have to collect additional observations and invest in parameter estimation, as no meaningful predictions are possible otherwise (Figure 7C). If the additional parameters turn out to be sloppy (Figure 7D), they may as well be omitted, as their inclusion does not advance predictions. Since it is often difficult to determine in advance how important a particular parameter will be, there is considerable scope for the production of realistic models if only for the purpose of demonstrating that a simpler model is appropriate too. Caution is necessary in these comparisons, as the addition or removal of parameters is likely to be accompanied by a change in the underlying non-linear logic of the simulation that can turn sloppy and stiff parameters into their respective opposite.

Computer simulations are a relatively new approach to generating knowledge and have not yet developed the maturity that comes from centuries of experimental or analytical theory work. While biologists are used to recognising good or poorly designed experiments and theoreticians know what can and cannot be proved, computer simulations are sometimes greeted with suspicion by both. To add to the confusion, computer scientists frequently talk about 'experiments' when they really mean a set of simulations. Here is not the space to review approaches to building quality simulations, but it is important to stress that there are many pitfalls and researchers in evolutionary systems biology have to develop the skills needed to avoid the many traps in the often interdisciplinary work of modelling and in the computer programming that goes with it [41,314,316-326].

The key challenges are to ensure that simulation results are free from bugs that affect the biological model and that the input parameters are relevant to the system that is being studied. This demands that simulations are routinely linked to simpler test cases from analytical theory (proved to be correct) and that model building includes a significant effort to determine realistic ranges for the parameters of the model. Simulations that are neither linked to the hard dry bones of analytical theory, nor to realistic biological parameters are the equivalent of a 'pile of rotten flesh' in the analogy of Table 3. Such poor work is responsible for much of the suspicion that 'non-simulation researchers' can have towards 'yet another simulation'.

Linking simulations to analytical theory is mostly manual work nowadays. However, the development of process algebras for simulating molecular reaction systems could prove crucial in this respect. Process algebras are formal languages that were designed to describe concurrent systems [327] and have recently been extended to allow the modelling of intracellular chemical reactions [328]. The beauty of a process algebra is in the independence of the model specification from its implementation. This opens the possibility of automatically translating the same model into a stochastic simulation on one occasion and into an ordinary differential equation system on the next [25]. These two independent implementations of a model might be used to assess their reliability.

The problem of parameter estimation is model specific, but can be greatly facilitated by statistical approaches, especially Bayesian statistics, which can handle arbitrary complex models with the help of enough computing power [311,312,329].

To build the models that bring us closer to mechanistic evolutionary systems biology, we need interdisciplinary approaches drawing from molecular biology, ecology, evolution, computer science, systems theory, analytical mathematics and statistics, combined with practical expertise in developing maintainable high-quality source code for models that is easy to debug.
